# A Scorecard for Information Synthesis in Multiple Experimental Conditions: Application to Bacterial Biofilm Matrix Transcriptomics

**DOI:** 10.1007/s00284-025-04435-3

**Published:** 2025-09-09

**Authors:** Mauro Nascimben, Lia Rimondini

**Affiliations:** Department of Health Sciences, Università del Piemonte Orientale UPO, Corso Trieste 15/A, 28100 Novara, Italy

## Abstract

A Python-scripted software tool has been developed to help study the heterogeneity of gene changes, markedly or moderately expressed, when several experimental conditions are compared. The analysis workflow encloses a scorecard that groups genes based on relative fold-change and statistical significance, providing additional functions that facilitate knowledge extraction. The scorecard reports highlight unique patterns of gene regulation, such as genes whose expression is consistently up- or down-regulated across experiments, all of which are supported by graphs and summaries to characterize the dataset under investigation. Four GEO datasets related to RNA-seq bacterial biofilm expression levels were independently analyzed for information mining through the functionalities of the software library. The scorecard identified and tracked, over time or experiments, genes meaningful for bacterial metabolism and survival in response to antibiotics, adjuvants, and biocompatible materials. Analyses detected factors and strategies to persist in the environment by bacterial aggregates, such as modifications in the binding affinity of penicillin-related proteins or ribosomal subunits, the development of alternative metabolic pathways, cell wall thickening, intracellular concentration of drugs reduced by efflux pumps, and enzymatic inactivation through hydrolyzation, phosphorylation, or adenylation.

**Supplementary Information:**

The online version contains supplementary material available at 10.1007/s00284-025-04435-3.

## Introduction

RNA-sequencing (RNA-seq), which is used to measure the quantity and sequences of RNA in a biological sample, provides a snapshot of gene expression and is usually accompanied by differential expression analysis which is a computational process to identify genes whose expression levels differ significantly between two or more conditions (e.g., treated vs. untreated, diseased vs. healthy). Interpreting differential expression (DE) data under several experimental conditions can be challenging and time-consuming due to the extensive information provided. Indeed, narrowing information in DE analysis allows researchers to focus on the most biologically relevant findings and reduce noise [1], aiding and supporting knowledge extraction. After preprocessing by removing genes with low expression, which are prone to technical noise, and quality control, a bioinformatic workflow typically uses strict cut-offs for p-values (usually adjusted to control the false discovery rate and reduce the chance of false positives) and fold-changes to represent data in volcano plots that focus on the most significantly DE genes over a log-log Cartesian plane [2, 3]. Volcano plots allow for easy visualization of both the magnitude and significance of changes, as shown in the web-app proposed by the authors of [4]. However, volcano plots could still be crowded because minor differences can be statistically significant if the sample size is large enough [5]. When the number of genes is very high, researchers may have difficulty filtering meaningful outcomes. For practical reasons, one could track only specific key entries suggested by experience or the literature. This experience-driven approach, which targets a preselected set of symbols, might hide relevant information arising from actual data [6]. Another limitation of volcano plots is that they show one condition per graph, which can be less informative in experiments involving multiple variables, treatments, or changes over time. When dealing with this type of experimental design, the authors in [7] suggest the use of a four-way plot: in this type of graph, two treatments or experimental conditions are compared based on their respective log fold-change with a control group, which is particularly valuable when multiple comparisons are made against a specific control or baseline sample. The x-axis in this visualization shows the log fold-change of “treatment 1” versus the control, whereas the y-axis displays the log fold-change of “treatment 2” versus the control. By setting a log fold-change threshold, this plot can be divided into distinct regions: the central areas represent genes with irrelevant modifications under both treatments compared with the control group, the outer upper-right and lower-left regions represent genes that are positively or negatively expressed under both conditions, and the upper-left or lower-right regions indicate genes that are highly expressed in one comparison and poorly expressed in the other. Nonetheless, a four-way plot can be prone to visual clutter: as the number of genes or data points increases, the plot becomes difficult to interpret; when the plot is saturated with points, its utility for identifying meaningful patterns decreases. Typical four-way plots are not filtered by statistical significance, which is essential in gene analysis. Due to a single fold-change threshold, subtle differences in gene expression may not be effectively highlighted, particularly when the range of expression levels is narrow or when the differences are significant only in a small subset of genes. Finally, there is no ability to merge information from the four-way plots.

### Identifying relevant genes using a scorecard

In general, bioinformatics scorecards [8, 9] are tools that are useful for quality control or tracking biological phenomena over a Cartesian plane by applying dimensionality reduction techniques [10, 11]. A “scorecard” of relative fold-change could be defined as a graphical method to visualize and compare changes in gene expression under different experimental conditions, by extracting common genes that are markedly or moderately up- or downregulated.

To overcome the limitations of four-way plots in DE analyses, a scorecard has been released as a free-to-use Python software library. The library incorporates additional features to facilitate the identification of patterns and interactions between assays. Compared to a canonical four-way fold-change plot, the scorecard introduces the creation of additional regions of interest in each Cartesian quadrant defined through two distinct fold-change thresholds. It filters the genes present on the graph based on their statistical significance. Moreover, the scorecard is complemented by additional features, including images, export options (in JavaScript Object Notation format, also known as JSON), and textual reports (in Excel or CSV table formats, as well as log files). For example, a specific function within the scorecard library could provide a comprehensive overview of how bioactivities vary under multiple conditions by merging all the information gathered from single scorecards. Another feature introduced by the scorecard library is the ability to highlight common changes across multiple experiments or isolated modifications, allowing for the understanding of condition-specific effects versus global changes. Moreover, most bioinformatic packages are designed in the R programming language; researchers who use Python as a medium for data analysis can integrate the proposed functions into their pipelines, supporting more sophisticated experimental questions and analyses, and enabling comprehensive data exploration. Indeed, researchers sometimes reduce the amount of transcriptomic data when analyzing results from multiple conditions by focusing their attention on specific, predefined genes. One of the goals of the scorecard is to bypass gene preselection, maintaining a data-driven feature extraction approach.

### Biofilm Data Analysis

Biofilms are complex microbial communities (bacteria, fungi, and archaea) that have been investigated to develop control strategies and combat biofilm-associated infections. Biofilm characterization via functional genomics or the evaluation of gene expression and regulation in bacteria can reveal genes’ functions and roles in biofilm formation, maintenance, and response to environmental changes [12]. In addition, understanding the proteins produced by biofilms and their post-translational modifications, or the metabolites formed by biofilms, which can indicate metabolic states and pathways, provides a detailed and multifaceted comprehension of the molecular and cellular processes in biofilms [13]. Furthermore, identifying genes involved in antibiotic resistance mechanisms, or how biofilms respond to environmental stress, including nutrient limitation, pH changes, and host immune responses, are findings that improve the understanding of their adaptive survival strategies [14]. Virulence factors such as pathogenicity and interaction with host tissues, genomic variability, and adaptive evolution are other aspects that should be recognized for biofilm control, to restrict initial adhesion and limit the propagation of extracellular polymeric substances that form the biofilm matrix [15].

The heterogeneity of biofilms in terms of cell types and nonuniform micro-environments may require stronger analysis procedures to handle their compositional and spatial asymmetries [16, 17]. Biofilm transcriptomic studies face numerous challenges, not only from a biological point of view but also from a technical and computational perspective. Massive RNA-seq datasets require significant computational resources and expertise, and in biofilm studies, due to the complexity of the samples, distinguishing noise from meaningful biological signals could be challenging [18, 19]. In addition, biofilms develop over time, and gene expression profiles change at different stages of biofilm formation (e.g., attachment, maturation, and dispersal). Capturing these dynamic changes requires longitudinal transcriptomic analyses, potentially complex and resource-intensive [20]. Although derived expression levels might be meaningful, some biofilm-associated genes may be poorly annotated or have unknown functions, making it difficult to interpret their roles in biofilm biology. Standardized protocols may be influenced by the variability in biofilm formation, even under controlled laboratory conditions. Differences in inoculum, growth conditions, and even minor environmental changes can lead to significant variability in biofilm structure and gene expression, making reproducibility challenging. Finally, wet-lab methods for extracting and purifying RNA may present issues due to the dense extracellular matrix and the physical structure of biofilms, which limit their efficiency [21].

### Aim of the Study

Four biofilm-related datasets were investigated using the scorecard functionalities for data-driven genetic information extraction, providing ad hoc interpretation and an enhanced understanding of gene behavior across experimental sessions. Using the scorecard, one could approach all the data obtained from transcriptome computations and extract relevant entries without preselecting key genes to produce biological insights; the scorecard will identify extreme (or moderate) variations scanning transcriptome symbols, shrinking the massive records usually encountered in RNA-seq data by screening both in terms of fold-change and statistical significance.

The application of the scorecard to clinically relevant strains of biofilms of *Staphylococcus aureus*, *Streptococcus mutans*, and *Enterococcus faecalis* will be discussed, to offer practical insights and demonstrate the potential inclusion of the scorecard in bioinformatics or data science pipelines based on the Python programming language.

## Materials and Methods

### Overview of Scorecard Software

**Fig. 1:**
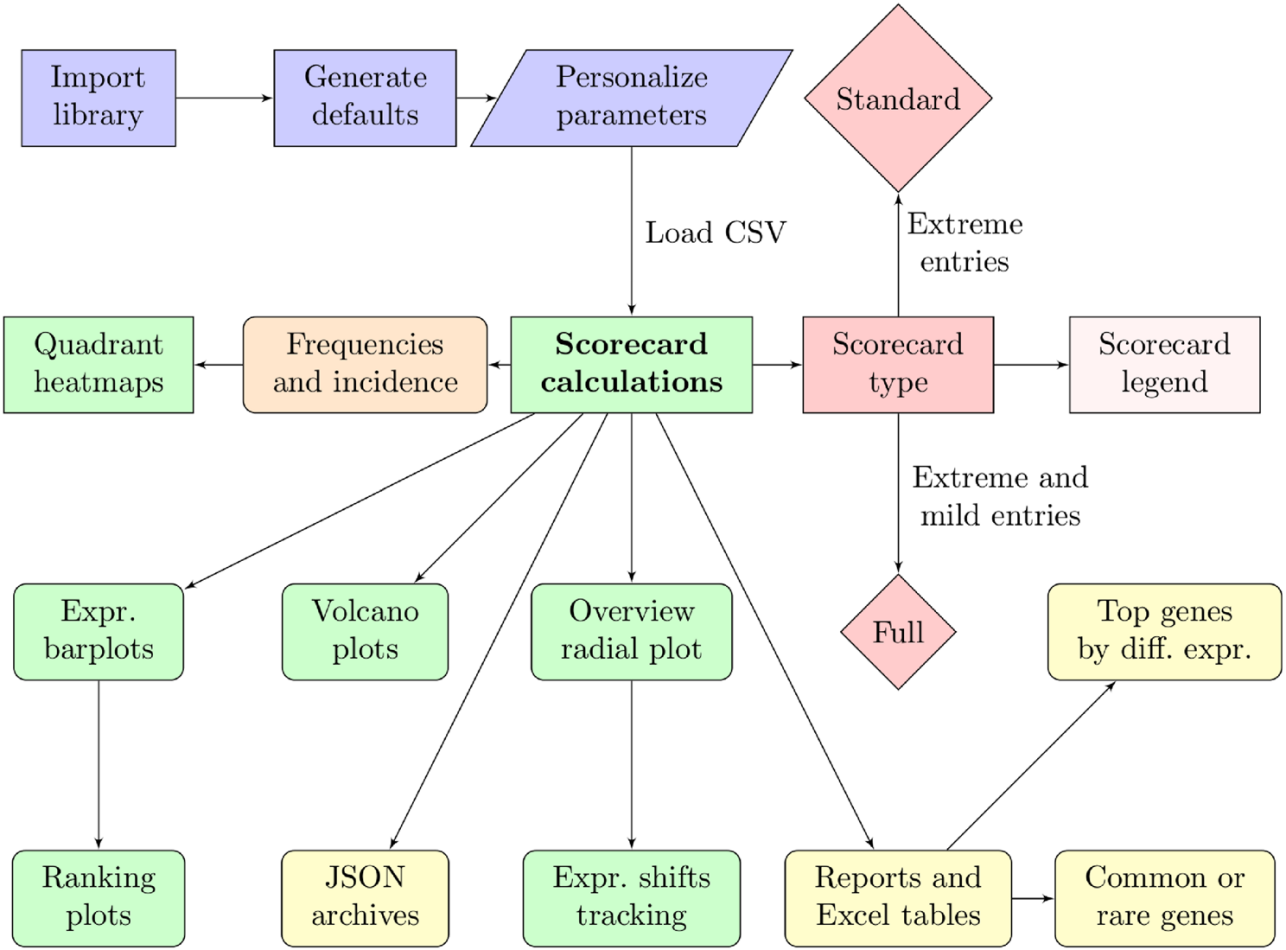
Illustration of the workflow offered by the Python library. Starting from the user-defined parameters and a CSV file containing the data series for each experimental condition, the scorecard’s library computes the scorecard while supporting functions create textual reports, graphs, and tables to aid data interpretation and JSON archives for information retrieval and further evaluation

Conceptually, the proposed scorecard is a sophisticated plot that merges four-way and volcano plots to create pairwise comparisons and classify shared genes into extreme or moderately changing groups on the basis of their actual expression profiles. By adjusting the two-fold-change thresholds, one can select extreme and moderate modifications compared with the standard four-way plot, which employs a single fold-change threshold. Additionally, the scorecard filters the results based on p-value differences, excluding the possibility that the observed differences are due to random chance and retaining only the relevant information for further study. The scorecard is enhanced by a series of functions that complete the analysis and aid in interpreting the data (Figure 1). Section 6 in the Supplementary Materials file outlines the operational steps for running a study using the software. Section 7 of the Supplementary Materials file contains a tutorial with the code needed to complete a synthetic dataset study.

By analyzing where a gene falls inside the scorecard, one can identify genes that show consistent or opposite regulation across experimental conditions or time points, scanning for genes with similar or contrasting expression to test scientific hypotheses. In complex experimental designs, visualizing several experimental conditions can be challenging due to the high number of volcano plots to analyze; however, a scorecard that filters relevant genes and categorizes the results from various related experiments can be particularly helpful. The scorecard software has been deployed on GitHub, as repository, free to download and install.

### Scorecard Regions of Interest

**Fig. 2:**
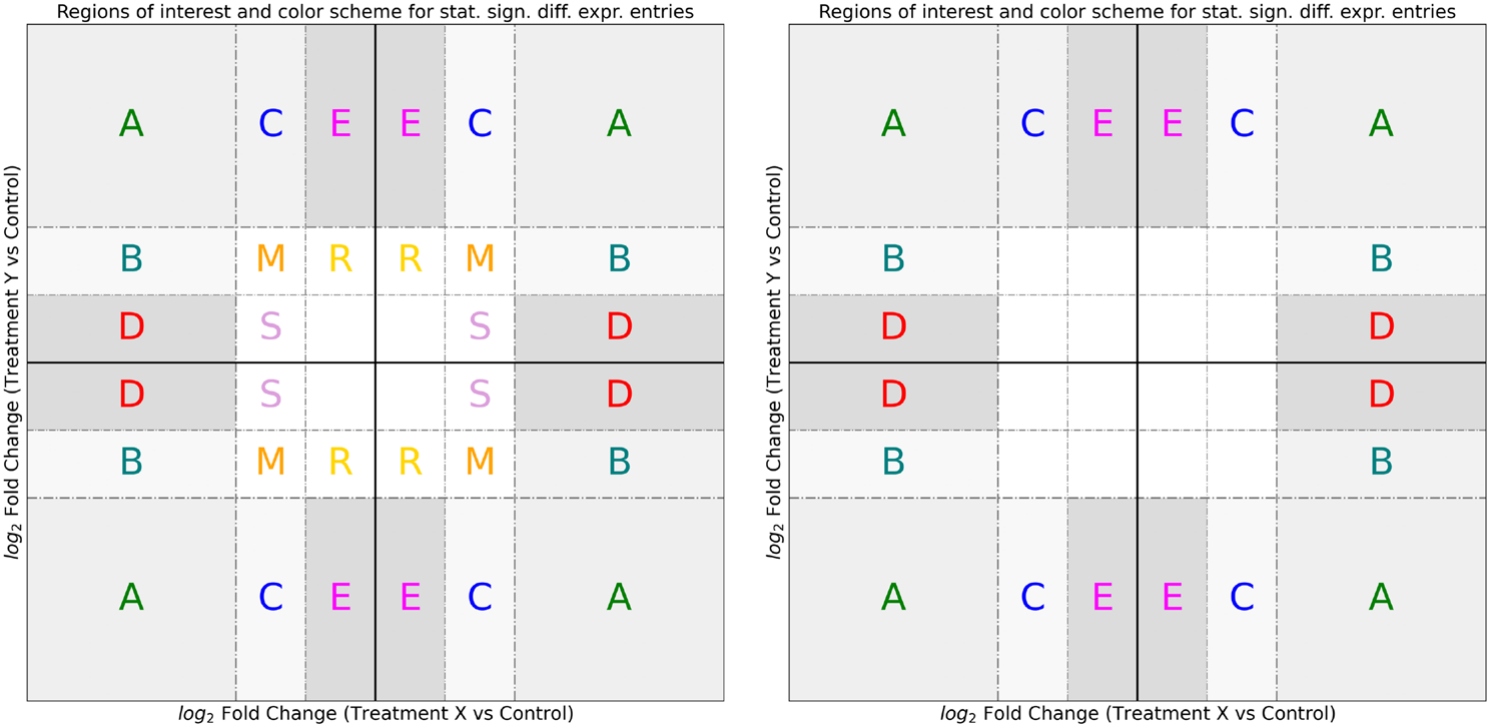
Regions of interest as identified by the scorecard: a specific identification letter and color are assigned to the regions of interest of each quadrant. Two different scorecards can be created: full scorecards with enhanced detail levels and standard (left and right panels); additionally, a classic four-way scatterplot could be computed (not included)

The scorecard’s detail level is customizable and includes additional functions to judge fluctuations in gene expression across the whole set of possible experimental conditions. Figure 2 shows three scorecard configurations that one could obtain by adjusting the fold-change thresholds to increase the level of detail. If the user inputs a single fold-change threshold, one can create a four-way plot conceptually similar to the one in the R library “ViDGER” [22]. Nevertheless, the scorecard can generate more accurate results than the four-way plot by tuning a second fold-change threshold and including statistical significance. In the scorecard, the regions of interest could include or exclude the moderately changing expression levels to highlight extreme values only or profile the relevant values into extreme and mild variations.

In the scorecard, two treatments are compared to a control group by plotting their respective log fold-changes: the x-axis represents the log fold-change of treatment X versus the control, while the y-axis represents the log fold-change of treatment Y versus the control. The plot is divided into distinct regions in each quadrant by applying log fold-change thresholds to the Cartesian plane of the scorecard. The central area contains genes with low fold-changes in both treatments compared to the control. When most data points lie in the central region, both treatments would have a comparable expression to the controls. The upper-right and lower-left quadrants represent positively or negatively expressed genes in both experimental conditions (i.e., **Q1** and **Q3**). Inside the upper-left and lower-right quadrants (second and fourth quadrants, abbreviated as **Q2** and **Q4**), one can find highly expressed genes in one condition but expressed at low levels in the other. Each scorecard region **A** contains the extreme values of the quadrants. Genes expressed markedly in one condition and mildly in another reside in areas **C** and **B**. Genes expressed exclusively in one condition could be found in regions **D** and **E**. From these regions, one can discern the full range of expression patterns strongly affecting the experimental variables. Another feature included in the scorecard library is the color code associated with each point belonging to each region of interest, which the user can edit: this unique color scheme is employed in all visuals (Supplementary materials section 5 declares the colors employed during the analysis of each dataset). Only genes or entries with p-values below the user-selected statistical threshold are reported inside the scorecard. The standard scorecard has two fold-change thresholds and hosts only statistically significant genes inside the outer regions of interest (right panel of Figure 2), whilst the full scorecard which reports all genes with extreme or moderate expression displaying statistical significance (left panel of Figure 2), is augmented with regions **M**, **R**, and **S** for mild changes.

If the precision level plays a crucial role in the investigation, the full scorecard (left panel of Figure 2) can be applied because it separates both extreme and moderately changing values into distinct groups. 

For small or medium-sized datasets, the suggested approach is to conduct two distinct analyses: one using the standard scorecard and another using the full scorecard, maintaining the same thresholds to capitalize on the distinct characteristics of the two scorecard types. For large datasets, one can focus on extreme variations from the standard scorecard, filter the information, and concentrate on specific genes with abnormal expression across experiments. During RNA-seq analysis, a common analysis method involves a dendrogram combined with a heatmap to display the clustering of samples by gene expression or the clustering of genes with similar expression patterns. The scorecard shows clusters of genes with similar expression patterns using the regions of interest, whereas sample grouping (for example, subdividing biological replicates into “treated” vs. “untreated” through the dendrogram) is replaced by experimental condition grouping, which provides a comprehensive assessment of all comparisons performed.

### Datasets Analysed by Applying the Scorecard

Biofilms are complex structures formed by bacterial cells encased in a matrix and adhering to solid surfaces. They play a significant role in chronic infections, particularly those related to medical devices and implants. Research shows that bacteria within biofilms may exhibit antibiotic resistance 100–1000 times greater than their free-floating counterparts [23]. Understanding the genetic profiles of biofilms is crucial for addressing infections. .Their unique tolerance and resistance characteristics, as well as the underlying molecular mechanisms, require detailed investigation to improve treatment strategies.

The following biofilm-related datasets were evaluated via the proposed software tool, and they were selected because they involve multiple experimental conditions:

Dataset 1: Staphylococcus aureus accounts for a significant number of invasive infections worldwide each year, posing challenges as it often exhibits resistance to antibiotic treatment. Antibiotic tolerance, defined as the capacity of bacteria to survive normally lethal concentrations of antibiotics, plays a crucial role in the failure of antibiotic therapies for *Staphylococcus aureus* infections. To investigate the mechanisms behind antibiotic tolerance, the dataset authors [24, 25] prepared biofilms of *S. aureus* subjected to various antistaphylococcal antibiotics and analyzed through a genome-wide TnSeq screen which compared *S. aureus* grown as a filter colony biofilm for 48 hours and exposed to 5 different conditions (four different antibiotics or left untreated) for another 48 hours.

Dataset 2: Methicillin-resistant *Staphylococcus aureus* (MRSA) poses a significant risk to human health. Instead of relying on the development of new antibiotics, which bacteria will eventually evolve resistance to, the Authors of the dataset [26] utilized antibiotic adjuvants to increase the effectiveness of existing antibiotics. The study investigated differential gene expressions across MRSA cultures subjected to various treatments: untreated, treated solely with oxacillin, treated exclusively with compound 8 or loratadine, and those receiving combined therapy with oxacillin and loratadine or compound 8. Each of the six treatment conditions was conducted with biological triplicates, and RNA libraries were prepared for sequencing via standard Illumina protocols. Gene expression levels were measured in fragments per kilobase of transcript sequence per million base pairs sequenced (FPKM) and averaged across biological triplicate samples.

Dataset 3: Recent advancements in RNA-sequencing have significantly improved the recognition of the cariogenic impacts of various carbohydrate sources on the bacterium *Streptococcus mutans*, which is the leading cause of dental caries in humans. The process of dental caries development is promoted by the ability of bacteria to ferment specific carbohydrates into organic acids, resulting in a reduction in pH in the oral environment and subsequent demineralization of tooth enamel. Furthermore, the formation of biofilms is essential to the progression of dental caries, which initiates and concludes with free-floating planktonic cells, and is characterized by several unique properties referred to as virulence factors. According to the literature, sucrose is identified as the most cariogenic carbohydrate due to its rapid metabolism, which enhances acid production and the formation of glucans that create distinct bacterial clusters [27]. The Authors of the dataset [28, 29] obtained the transcriptomic profiles of *Streptococcus mutans* grown in planktonic culture under conditions of preferred and nonpreferred carbohydrates, as well as during fasting. The expression profiles of biofilm growth in tryptone soy broth medium in contact with glucose, sucrose, lactose and xylitol for six hours at $37^{\,\circ }$C with 5% CO_2_.

Dataset 4: Antibiotic resistance in clinically relevant bacteria, along with their ability to form biofilms on medical and technical devices, poses a significant challenge for effective and long-term decontamination in healthcare settings, necessitating the development of new antimicrobial materials. The dataset [30, 31] examined the impact of AGXX^®^, a broad-spectrum antimicrobial surface coating made of microgalvanic elements formed by silver and ruthenium, on the transcriptome of the nosocomial pathogen *Enterococcus faecalis*. The stainless-steel carrier meshes (V2A: DIN ISO 1.4301) were coated with AGXX^®^, or silver, or left untreated. The three metal samples were exposed to *Enterococcus faecalis* strain 12030 [32] for 3, 6, 12, 24, 60 or 90 minutes at $37^{\,\circ }$C with constant agitation. Length-normalized confidence interval FPKM (Fragments Per Kilobase of exon per Million fragments mapped) values were obtained from RNA-sequencing.

The analyses through the scorecard involved the preprocessed data released by the original authors of the four datasets in tabular format (either Excel or CSV files).

## Results

Analyses of biofilm-related expression levels in the four datasets are detailed below to demonstrate the usage of the scorecard and its application to real-world data. All the datasets contain several experimental conditions that will be compared via the scorecard; the numerical experiments were computed on commodity hardware (specifications in Supplementary material section 3). Supplementary material file section 4 contains general information produced by the scorecard library for dataset 1 in Table SM4, Table SM7 for dataset 2, Table SM5 for dataset 3, and Table SM6 for dataset 4.

### Using the Scorecard on Dataset 1

The dataset encompasses a comprehensive genome-wide analysis of transposon insertion sequences, comparing *Staphylococcus aureus* (SAUSA300) under five different conditions (four antibiotics or untreated) for 48 hours. The antibiotics used were Vancomycin 400 mcg/ml (VAN), Linezolid 20 mcg/ml (LZD), Ceftaroline 20 mcg/ml (CPT), and Delafloxacin 9 mcg/ml (DEL). The $log_{2}$ fold-changes between antibiotic-exposed biofilms and the nonantibiotic controls were calculated via the resampling method in TRANSIT with beta-geometric normalization and correction for multiple comparisons using the Benjamini-Hochberg procedure. In this dataset, six comparisons were made applying the scorecard and setting fold-change thresholds between 2 and 3 (that is, a multiplication factor of 1.5) and a significance level of $p \le 0.05$, as summarized in Table SM4. Table 1 lists the genes identified in all regions of interest for each quadrant of the full scorecard (abbreviated as Q1, Q2, Q3, Q4, plus the letter denoting the region of interest as shown in Fig. 2). The entries in Table 1, show that the comparisons involving VAN mainly identified markedly down-regulated genes in Q3; in contrast, the other antibiotic-related genes were markedly over-expressed (SAUSA300_RS03525, SAUSA300_RS04560, and SAUSA300_RS08475 were the most represented ones). The lower-left quadrants of the standard scorecard involving comparisons with VAN are shown in Figure SM3: the identified genes are highly underexpressed for VAN without showing a relevant modification in the other antibiotics.

**Table 1:** Number of extremely and moderately varying genes on Dataset 1 using the full scorecard

X,Y axes	Q1 A	Q1 B	Q1 C	Q1 M	Q1 S	Q1 R	Q3 D	Q3 M	Q3 S	Q3 R
CPT LZD	2	1	0	3	4	4	0	0	0	4
DEL CPT	2	0	1	3	4	2	0	0	1	0
DEL LZD	4	0	0	5	4	2	0	1	0	4
VAN CPT	0	0	0	0	0	0	7	0	4	0
VAN DEL	0	0	0	0	0	0	4	0	3	1
VAN LZD	0	0	0	0	0	0	6	1	5	1

VAN is a glycopeptide antibiotic that inhibits cell wall synthesis through osmotic lysis, which subsequently induces a stress response in bacteria [33, 34]. For instance, SAUSA300_RS10935 (accessory gene regulator AgrB) was greatly differentially expressed in all comparisons involving VAN, probably for its participation in the synthesis, processing, and export of the autoinducing peptide (AIP), which aids quorum sensing in *S. aureus* by fine-tuning its response to environmental conditions, shifting from an adhesive, colonization-focused phenotype to an invasive, toxin-producing phenotype [35]. Another gene with similar behavior is SAUSA300_RS10950 (LytTR family DNA-binding domain-containing protein) for cell wall maintenance, biofilm formation, or dispersal. The VAN vs. CPT and LZD, activated gene SAUSA300_RS11145 (type II toxin-antitoxin system PemK/MazF family toxin) displaying various biological functions in *S. aureus* pathogenicity, whereas, for LZD vs. VAN, the scorecard detected SAUSA300_RS11125 (anti-sigma B factor RsbW) linked to the formation of persister cells and increased antibiotic tolerance.

From the log files generated by the scorecard, it is possible to count the occurrence of each single entry belonging to the areas of interest of each quadrant for any experimental condition. For example, the extracted symbols SAUSA300_RS03455 (auxiliary protein GraX/ApsX), SAUSA300_RS03475 (ABC transporter permease VraG), SAUSA300_RS06820 (bifunctional lysylphosphatidylglycerol flippase/synthetase MprF), and SAUSA300_RS11135 (PP2C family protein-serine/threonine phosphatase) are all negatively enhanced in VAN compared with the other antibiotics. The entry SAUSA300_RS03465 (histidine kinase GraS/ApsS) has the same behavior but is markedly downregulated in VAN compared with CPT and LZA only. Serine–threonine kinases (STKs) are recognized for their role in the development of antibiotic resistance in bacteria: these kinases are part of a group of enzymes that phosphorylate serine and threonine residues on proteins, thereby regulating protein function [36].

Another experimental factor was CPT, a broad-spectrum antibiotic capable of combating methicillin-resistant *Staphylococcus aureus*. It works by inhibiting bacterial cell wall synthesis by attaching penicillin-binding proteins, which are essential for cross-linking peptidoglycan chains in the bacterial cell wall. Without proper cell wall synthesis, bacteria become unstable and eventually die [37]. Ceftaroline has been shown to penetrate the extracellular matrix of biofilms, allowing it to reach and affect the bacteria embedded within, and has been demonstrated to be active against sessile bacteria, reducing the overall bacterial load within biofilms [38]. On CPT, compared to DEL and LZD, the entry identified by the scorecard settled in the first quadrant, indicating extreme overexpression for SAUSA300_RS08475 (shikimate dehydrogenase); additionally, CPT, DEL, and LZD have a common overexpression falling in the first quadrant for SAUSA300_RS03525 (DUF402 domain-containing protein) and SAUSA300_RS04560 (NAD(P)/FAD-dependent oxidoreductase).

**Fig. 3:**
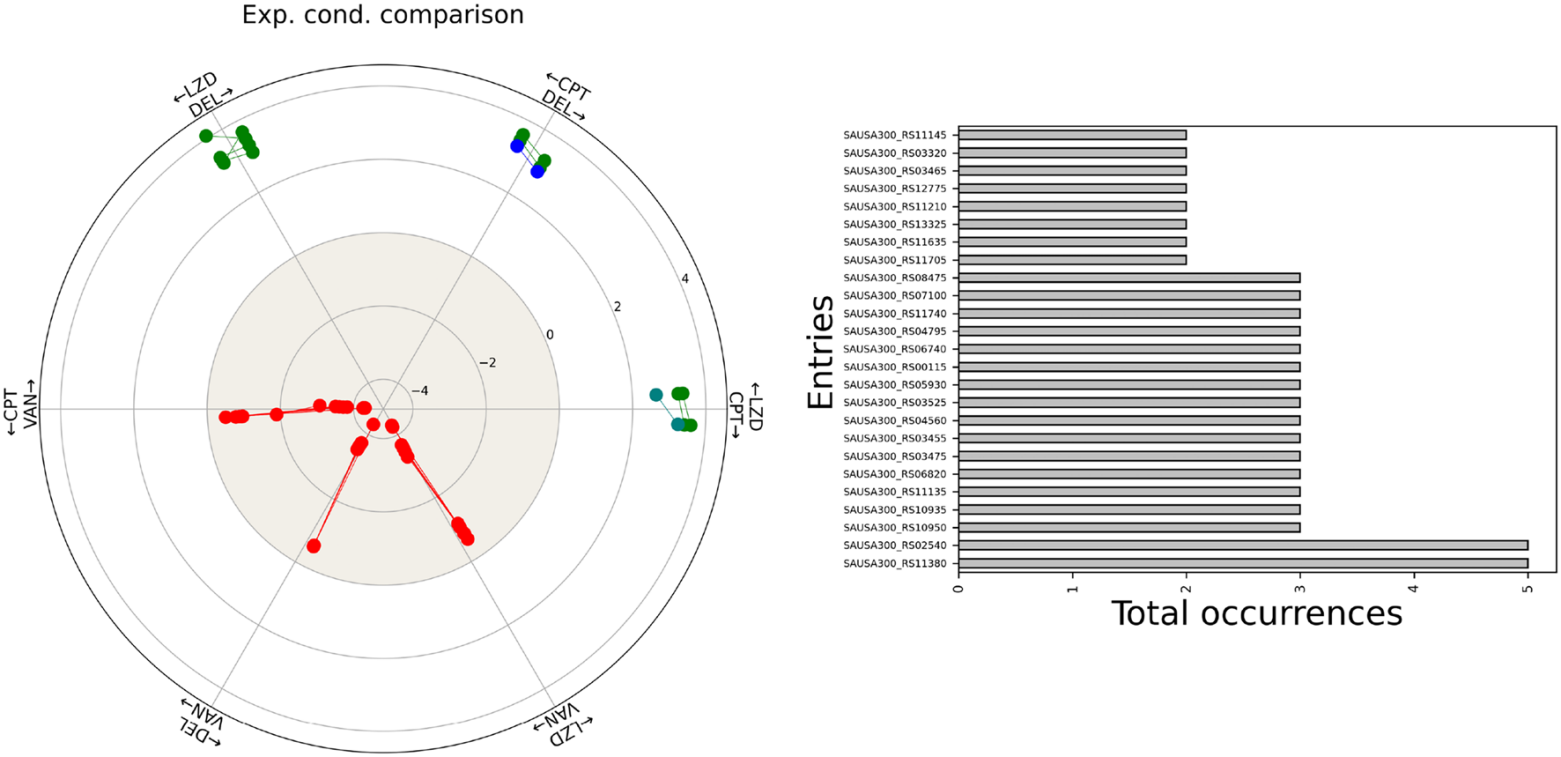
Left image contains genes identified from all comparisons via the standard scorecard on a single graph (dataset 1). The bar plot on the right shows gene occurrences when the full scorecard is applied (dataset 1)

To judge all the experimental conditions reported in Table 1, there is a specific function included in the software library that produces a general visualization of all comparisons, displaying the magnitude of the variations in terms of fold-change (Figure 3, left panel). In this picture, the color code matches that of each region of interest of the scorecard. Additionally, two experimental conditions are shown on the left or right of each radius: the variations in the fold-change are represented by the dots, and the line connecting them depicts the variation in the expression values encountered. Observing the radial plot, differences in gene expression involving VAN can also be appreciated in their magnitude: the most prominent differences are for the genes identified by comparing VAN to the other antibiotics, inside the negative area (larger distances between dots).

The right part of Figure 3 examines the occurrence of single genes within each region of interest, applying the full scorecard, which encompasses moderately modified genes. The genes SAUSA300_RS11380 (uracil phosphoribosyltransferase), and SAUSA300_RS02540 (pur operon repressor) were found 5 times during the experimental comparisons (CPT DEL, CPT LZD, DEL LZD, VAN DEL, VAN LZD for SAUSA300_RS11380, and CPT LZD, DEL LZD, VAN CPT, VAN DEL, VAN LZD for RS02540); despite being frequently activated, the variations fell on the intermediate areas of interest of the scorecard, implying moderate modifications. The scorecards could also identify groups of distinctly active genes for specific experimental conditions. The standard scorecard pinpointed SAUSA300_RS10185 (two-component system response regulator VraR), and SAUSA300_RS10190 (sensor histidine kinase) in DEL vs. LZD, SAUSA300_RS03470 (ABC transporter ATP-binding protein VraF) and SAUSA300_RS03930 (GGDEF domain-containing protein) for VAN vs. CPT, and SAUSA300_RS11120 (RNA polymerase sigma factor SigB) in VAN vs. LZD. The two-component system VraSR in *Staphylococcus aureus* regulates the bacterial response to cell wall damage and antibiotic stress, particularly from cell wall-active antibiotics like vancomycin, daptomycin, and $\beta$-lactams: VraR is the response regulator, and VraS is the sensor kinase. Upon sensing external stress (such as antibiotic-induced cell wall damage), VraS (the sensor kinase) phosphorylates VraR, activating it: once phosphorylated, VraR can bind to DNA and control the transcription of genes associated with cell wall repair, the stress response, and antibiotic resistance, including genes that encode enzymes involved in peptidoglycan synthesis and turnover, making the bacterium more resilient in clinical settings [39]. Regarding SAUSA300_RS03470 and SAUSA300_RS03930, the first refers to the VraF protein which is likely involved in providing energy for hydrolysing ATP to transport molecules across the cell membrane, whereas the latter pertains to the GGDEF domain-containing protein that is involved in the synthesis of cyclic di-GMP (c-di-GMP), a second messenger that regulates various bacterial processes, including biofilm formation, motility, and virulence. Specific to LZD vs. DEL and CPT are markedly differentially expressed genes SAUSA300_RS13325 (MFS transporter) for the efflux of antibiotics and other toxic compounds out of the bacterial cell, contributing to multidrug resistance and indirectly to biofilm formation, and gene SAUSA300_RS05930(dihydroorotase), part of the *de novo* pyrimidine biosynthesis pathway for nucleic acid production [40].

### Using the Scorecard on Dataset 2

The curators of the dataset studied *Staphylococcus aureus* cultures untreated (Un) as a baseline, treated with oxacillin only (Ox), treated with compound 8 (Cmpd8, brominated carbazole) or loratadine (Lor) alone, or cotreated (noted with an underscore) with oxacillin and loratadine or compound 8. The expression values were obtained from RNA-seq of cultures of treated methicillin-resistant *Staphylococcus aureus* (SA) USA100 (Pulse-Field Gel Electrophoresis classification system), strains historically associated with nosocomial infections [41]. The fold-change thresholds were set to 2 and 3 with a significance threshold of 0.05 (from Table SM7). Table SM1 extracted the experimental conditions where one region of the scorecard had at least 10 entries, whereas Table SM2 presents scorecard entries obtained by fixing the second term of comparison on each axis. Exploring Table SM1, Lor_Ox co-treatment was included in nearly all comparisons, except Ox vs. Compd8 related to Compd8_Ox vs. Ox. Undeniably, using Lor to support antibiotics such as oxacillin for bacterial biofilm eradication is an established option [42], with dataset 2 aligning with the trending literature.

**Fig. 4:**
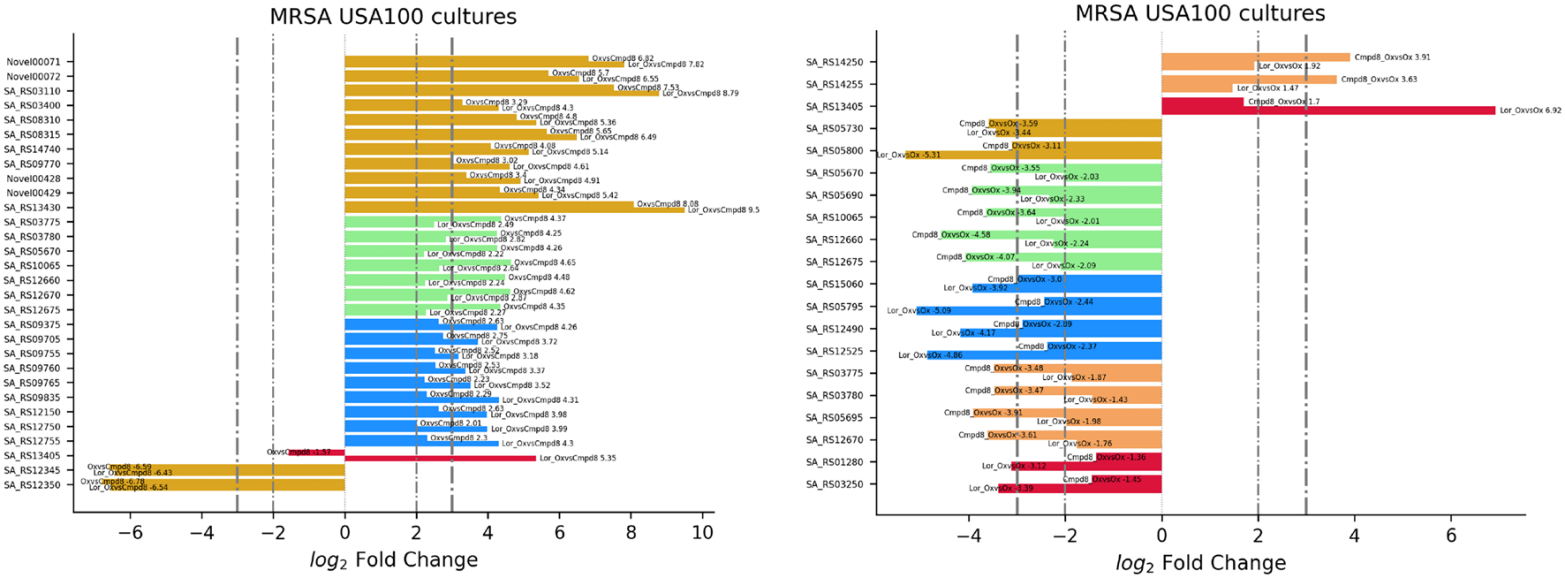
On the right: Expression levels of oxacillin vs. compound 8 and cotreatment with oxacillin and loratadine compared with those of compound 8 (dataset 2). The color scheme of the bars follows the arrangement selected by the user for the scorecard. On the left: Expression levels of the Lor_Ox cotreatment vs. Ox and Compd8_Ox cotreatment vs. Ox (dataset 2)

The last row of Table SM2 contains several genes that are extremely overexpressed in the Ox vs. Un and Lor_Ox cotreatment groups compared to the control. The same frequent bioactivity was found for Lor vs. Un and Lor_Ox vs. Un. Another interesting finding from Table SM2 is the activation of genes for Compd8_Ox vs Un and Lor_Ox vs. Un, which could serve as drug-specific markers. Additionally, the number of genes present inside each quadrant and region of interest of the standard scorecard was high in the oxacillin (Ox) or cotreatment with oxacillin and loratadine (Lor_Ox) vs. compound 8 (third row of Table SM2), probably defining Lor-specific bioactivities. Focusing on this last experimental relationship, the expression level visualization of each gene identified by the scorecard was plotted as bar plot through a specific function included in the scorecard library (Figure 4, left panel): gene SA_RS13405 displayed a diverging expression pattern, whilst distinct subgroups had a major expression level for Ox (light green bars), or Lor_Ox (blue bars).

The effect of antibiotic reinforcement by adjutant drugs could be assessed by interpreting Ox cotreated with Compd8 vs. Ox and Ox cotreated with Lor vs. Ox (Figure 4, right panel). In this comparison, the gene SA_RS13405, which is known to be bioactive on the cell wall, was identified extremely overexpressed in response to Lor_Ox cotreatment (6.92) compared with that in response to Compd8_Ox cotreatment (1.7). In contrast, the genes SA_RS14250 (peptide resistance ABC transporter ATP-binding subunit VraD) and SA_RS14255 (peptide resistance ABC transporter permease subunit VraE) were markedly overexpressed when Ox was combined with Compd8 rather than Lor.

Considering the experiment matching Lor_Ox co-treatment vs. no treatment to Compd8_Ox co-treatment vs. no treatment (Figure SM2, left panel), a subgroup of genes was markedly over-expressed in Lor_Ox cotreatment but nearly silent in Compd8_Ox (light orange positive bars in Figure SM2), which could be a genetic signature of Lor adjunction. Members of this subgroup were SA_RS09375 (peptidylprolyl isomeras), SA_RS09760 (sensor histidine kinase), SA_RS09765 (cell wall-active antibiotics response protein LiaF), and SA_RS09835 (23S rRNA (uracil(1939)-C(5))-methyltransferase RlmD). A similar pattern of expression was observed, but Compd8_Ox was slightly higher, in SA_RS01210 (M23 family metallopeptidase), Novel00071 (PF07690:Major Facilitator Superfamily), and SA_RS13405 (fatty acid efflux MMPL transporter FarE) as shown by the green positive bars in Figure SM2 (left side). Inspecting the same figure, the expression patterns were extremely downregulated in response to Lor_Ox cotreatment and nearly inactive in reaction to Compd8_Ox cotreatment for gene SA_RS12520 (respiratory nitrate reductase subunit gamma), intriguingly linked to specific *S. aureus* mechanisms of survival [43]. The blue bars in the left part of Figure SM2 could be considered descriptors of the Compd8_Ox cotreatment because of the marked expression of SA_RS12660 (immunoglobulin-binding protein Sbi), SA_RS14250 (peptide resistance ABC transporter ATP-binding subunit VraD), and SA_RS14255 (peptide resistance ABC transporter permease subunit VraE) after Compd8_Ox cotreatment rather than after Lor_Ox cotreatment, which evoked moderate expression in all three genes. Another possible experimental situation that merits attention is the effect of Lor’s adjunction to antibiotic Ox against the treatment with Lor only, both versus untreated samples (Figure SM2, right panel). The red bars, which differ due to Ox, represent the genes SA_RS01210 (M23 family metallopeptidase) and SA_RS03400 (M50 family metallopeptidase), altogether with genes SA_RS09705 (monofunctional peptidoglycan glycosyltransferase SgtB), SA_RS09760 (sensor histidine kinase), SA_RS09765 (cell wall-active antibiotics response protein LiaF), SA_RS12750 (glycerate kinase), and SA_RS12755 (membrane protein).

The software library could also highlight genes specifically active in one comparison and silent in all the others: Table 2 contains those identified by the standard scorecard. These genes could have a specific role related to the active biological processes or pathways under that condition, or the gene is part of a regulatory network that responds to particular stimuli. Considering that if a gene’s expression is uniquely tied to a certain condition, it could serve as a biomarker for that condition because it could be essential for the survival, adaptation, or functioning of the bacteria under that condition.

**Table 2:** Genes identified by the scorecard as being biologically active in only one experimental condition (dataset 2)

X-axis	Y-axis	Quadr. ROI	Symbol
Cmpd8vsLor	OxvsLor	Q1 A	RS12530
Cmpd8vsLor	OxvsLor	Q1 A	RS12545
Cmpd8vsLor	OxvsLor	Q1 C	RS12550
Cmpd8_OxvsOx	Lor_OxvsOx	Q3 B	RS05690
Cmpd8_OxvsOx	Lor_OxvsOx	Q3 D	RS05695
Cmpd8_OxvsOx	Lor_OxvsOx	Q3 E	RS01280
Cmpd8_OxvsOx	Lor_OxvsOx	Q3 E	RS03250
Lor_OxvsUn	Cmpd8_OxvsUn	Q1 A	RS05000
Lor_OxvsUn	Cmpd8_OxvsUn	Q3 A	RS06495
Lor_OxvsUn	Cmpd8_OxvsUn	Q3 B	RS14495
Lor_OxvsUn	Cmpd8_OxvsUn	Q3 B	RS15065
Lor_OxvsUn	Cmpd8_OxvsUn	Q3 B	RS12505
OxvsCmpd8	Lor_OxvsCmpd8	Q1 A	RS03400
OxvsCmpd8	Lor_OxvsCmpd8	Q1 A	Novel00428
OxvsCmpd8	Lor_OxvsCmpd8	Q1 A	Novel00429
OxvsCmpd8	Lor_OxvsCmpd8	Q1 C	RS09705
OxvsCmpd8	Lor_OxvsCmpd8	Q1 C	RS12150
OxvsCmpd8	Lor_OxvsCmpd8	Q1 C	RS12750
OxvsCmpd8	Lor_OxvsCmpd8	Q1 C	RS12755

The underscore on the X-axis and Y-axis columns indicates cotreatment

Alternatively, all scorecards from all combinations of the experimental conditions could be compressed into a single graph reporting the relative positioning of a gene across experiments; for example, the most frequently encountered one is gene SA_RS13430 which appeared on 65 scorecards (left panel of Figure SM3). Other recurring genes displaying extreme behavior across scorecards were SA_RS03110 (appearing on 63 scorecards, right panel of Figure SM3), SA_RS13405 (60 scorecards), SA_RS08315 (56 scorecards), Novel00071 (45 scorecards), and SA_RS12660 (39 scorecards) as shown in Table 3.

**Table 3:** Frequencies of *Staphylococcus aureus* genes across all possible 105 combinations of the experimental conditions in dataset 2

Gene	Frequency	Notes
RS13430	65	Cell wall inhibition responsive protein CwrA
RS03110	63	C1q-binding complement inhibitor VraX
RS13405	60	Fatty acid efflux MMPL transporter FarE
RS08315	56	PF16284:Domain of unknown function (DUF4930)
Novel00071	45	PF07690:Major Facilitator Superfamily
RS12660	39	Immunoglobulin-binding protein SBI, PF02216

Gene SA_RS03110 is linked to VraX, which is a protein secreted by *Staphylococcus aureus* whose deletion decreases the pathogenesis of the bacteria; for instance, overexpression of VraX might be linked to attempts of *S. aureus* to survive [44], whereas SA_RS13430 in *S. aureus* has a key role in stimulating biofilm formation, also associated to virulence modulation caused by hemolytic activity fluctuations in the presence of SA_RS13430 mutations [45]. Gene SA_RS12660, found in several *S. aureus* strains, including methicillin-sensitive and -resistant strains, could help mediate bacterial evasion through a futile fluid-phase consumption mechanism [46, 47].

Finally, a comprehensive overview of all experimental conditions can be achieved by considering the descriptions of the genes located in the outer regions of the scorecards, which may help identify recurring biological processes from annotations. Frequent terms were “phenol-soluble modulin” (found 70 times in 1126 gene descriptions), “nitrate reductase” (60 times in 1126 gene descriptions), “PF07968:Leukocidin/Hemolysin toxin family” (appearing 62 on 1126 gene descriptions), “ABC transporter” (encountered 82 times), and “crwA (65 times); while “crwA” should be about the stress caused by antibiotics that target the cell wall, ABC are transporters of substances across cellular membranes also functioning as efflux pumps. A slightly less recurring keyword was “metallopeptidase” (36 times) and “complement convertase inhibitor” (23 occurrences), both as Efb (extracellular fibrinogen-binding protein) and Ecb (extracellular complement-binding protein).

### Using the Scorecard on Dataset 3

Dental caries is a multifactorial disease caused primarily by the interaction between dietary carbohydrates, particularly sugars, and the bacterial biofilm (plaque) that forms on teeth [48]. The RNA-seq dataset considered transcriptomic modifications of *Streptococcus mutans* (SMU) depending on different dietary sources of carbohydrates: glucose (Glu), sucrose (Suc), lactose (Lac), and xylitol (Xyl) [29]. Among cariogenic carbohydrates, sucrose is considered the most cariogenic because of its unique role in biofilm formation and acid production. Oral bacteria, particularly *Streptococcus mutans* and *Lactobacillus* species, can ferment sugars, producing acids as metabolic byproducts [49]. Bacteria such as *Streptococcus mutans* utilize sucrose (Suc) to produce extracellular polysaccharides (glucans) through the action of glucosyltransferase enzymes. These glucans increase bacterial adherence to the tooth surface and contribute to the matrix of the biofilm, making it more robust and challenging to remove. As the biofilm matures and thickens, the environment becomes more conducive to acid production and retention, creating localized acidic conditions that can demineralize the enamel.

Initially, the scorecard parameters were set as for datasets 1 and 2; however, owing to the high number of genes included, the thresholds were modified to detect fewer outliers (Table SM5) using fold-change thresholds of 2 and 4 (aka, multiplication factor of 2) and p$\le$0.001. In total, six comparisons were considered by employing the standard scorecard: the number of genes identified within each region of interest is in the left part of Figure SM4. Observing the number of genes appearing on the heatmap, the comparisons involving Lac vs. Suc, and Suc vs. Xyl involved 37 and 34 common genes with opposite expression (belonging to quadrants IV and II). The genes falling in the regions of interest of the scorecard displaying opposite behavior for Suc compared with Xyl and Lac could be studied to determine genetic signatures associated with this carbohydrate, which has high cariogenic potential because it produces significant modifications in acid–base equilibrium affecting biofilm homeostasis [50]. Also, the evaluation of Lac and Glu pinpointed 23 genes following a reversed pattern of expression (belonging to quadrant IV). Instead, comparing Lac vs. Xyl revealed 33 genes whose expression moved in the same direction (overexpression in quadrant I), whereas Glu vs. Suc involved 24 downregulated genes (quadrant III).

The circular arrangement of all comparisons and expression values for each identified gene is shown in Figure SM5. This graph suggests that the most considerable differences in expression values arise from the comparisons involving Suc vs. Xyl, Suc vs. Lac, Glu vs. Lac, and Glu vs. Xyl. The relative differential expression in Lac vs. Xyl is small (short distance between points on the two sides of the Lac vs. Xyl radius), as in Glu vs. Suc, but on the opposing side. For example, the aguA (Putative agmatine deiminase) gene is highly overexpressed in Lac (4.94 f.c.) contrasted with Glu (1.98 f.c.), but it had a similar intensity value compared with Xyl (aguA for Lac was 4.34 and for Xyl was 4.32 in fold-change terms). The primary function of agmatine deiminase is to deiminate agmatine, removing the guanidinium group, producing N-carbamoylputrescine, and regulating the polyamine levels within bacterial cells, which helps the organism use agmatine as a carbon and nitrogen source [51, 52]. Another interesting behavior belongs to gene otcA (Putrescine carbamoyltransferase), identified by the scorecard as extremely active under nearly all experimental conditions except for Suc vs. Glu. This gene could be linked to aguA activity.

**Fig. 5:**
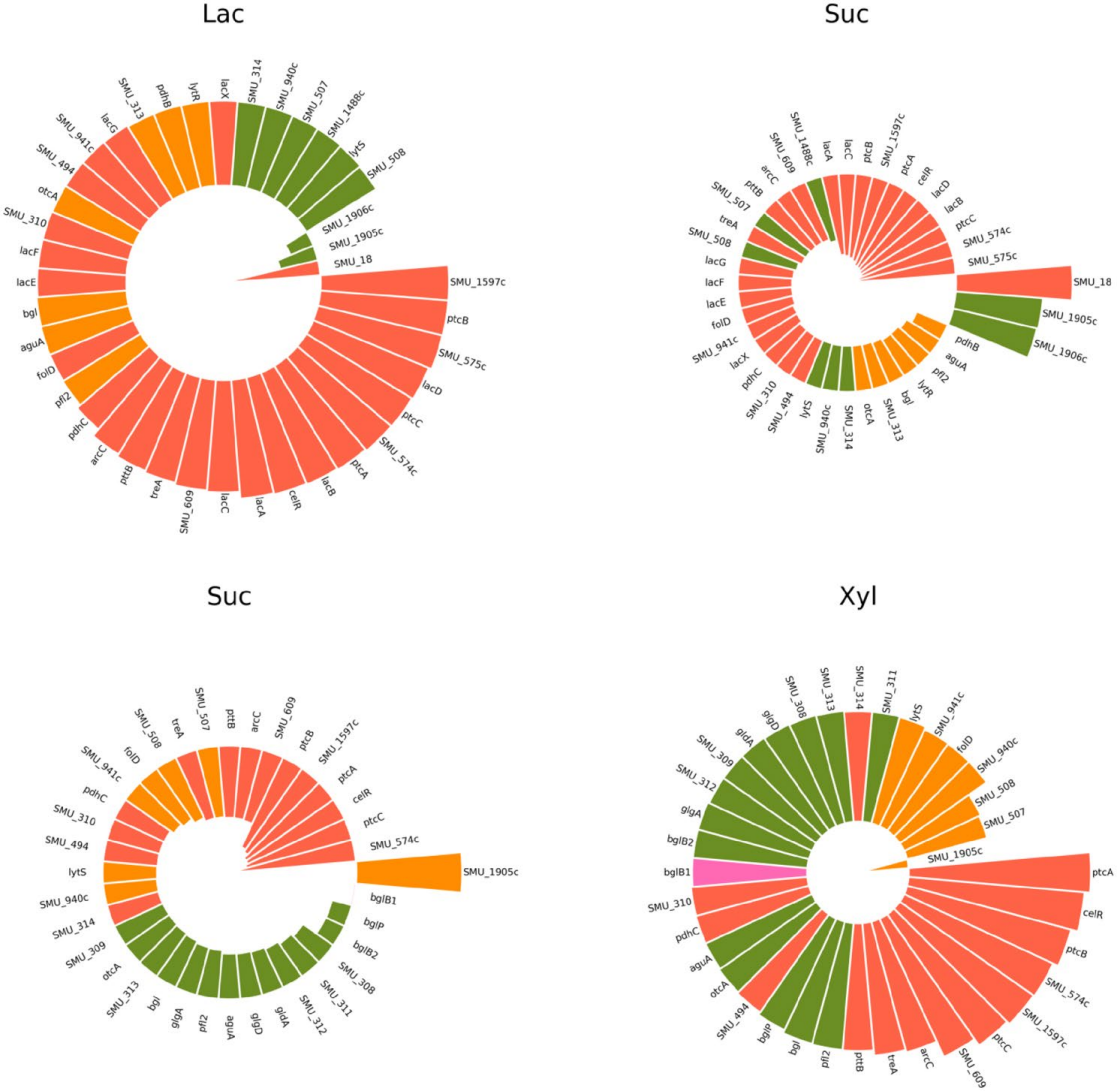
Patterns of opposite genetic expression for *Streptococcus mutans* as identified by the scorecard for Suc vs. Lac (paired ranking graphs in upper panel), and Suc vs. Xyl (lower panel) during dataset 3 analysis

Figure 5 shows the peculiarity of the expression levels found in Suc vs. Lac and Suc vs. Xyl with several genes exposing opposite regulation. Conversely, Suc vs. Glu had genes that were mainly expressed in the same direction, except for arC (Carbamate kinase). A gene highly expressed at opposite levels for both Suc vs. Lac and Suc vs. Xyl is SMU_574c, encoding a cell wall (membrane) protein [53]. In Suc vs. Lac specifically, the gene SMU_575c also displayed the same opposite behavior; both SMU_574c and SMU_575c are linked to the operon lrgAB, which encodes proteins that are involved in regulating cell death and lysis, particularly in response to environmental stress conditions. This operon plays a crucial role in enabling bacteria to survive under adverse conditions and contributes to biofilm formation and virulence [54, 55]. Other genes antithetically regulated in the bar plots of both Suc vs. Lac and Suc vs. Xyl are genes SMU_310 (sorbitol operon activator), SMU_494 (putative transaldolase), and SMU_609 (cell wall protein precursor). *S. mutans* employs a global regulatory mechanism known as carbon catabolite repression (CCR), which prioritizes the use of certain sugars (including glucose and sucrose) over others (like sorbitol). The CCR ensures that when a preferred carbon source such as sucrose is available, the expression of genes involved in the metabolism of less preferred sugars (such as those in the sorbitol operon) is repressed [56, 57]. Sucrose, a favored sugar for *S. mutans*, triggers CCR, leading to the downregulation of the sorbitol operon; this means that sucrose can inhibit the activation of the sorbitol operon by repressing the sorbitol operon activator. Moreover, gene SMU_494 was discovered to be involved in the membrane-associated Cid/Lrg system by [58], which is encoded by two paralogously related dicistronic operons, lrgAB (SMU_574c–SMU_575c) and cidAB. Instead, gene SMU_609 appeared to be part of the cysteine metabolism regulator CysR network, which was previously found to be significantly upregulated in response to the carolacton metabolite [59]. The scorecard also detected a subset of genes markedly expressed only in the Lac vs. Suc comparison: the genes lacA (galactosidase acetyltransferase), lacB (galactose-6-phosphate isomerase subunit LacB), lacC (tagatose-6-phosphate kinase), and lacD (tagatose-1,6-bisphosphate aldolase) involved in lactose catabolism mediated by lac operon were all overexpressed in Lac and underexpressed for Suc.

For genes found to be extremely active in all six comparisons (right panel of Figure SM4), the genes ptcA (cellobiose-permease IIa component, phosphotransferase enzyme II, A component), ptcB (cellobiose-specific IIB component), and ptcC (permease IIC component) should be connected to $\beta$-glucoside metabolism [60] as should celR (putative transcriptional regulator possible antiterminator), a transcriptional regulator typically involved in the regulation of genes responsible for the metabolism of cellobiose and related sugars [61]. The genes ptcA, ptcB, and ptcC encode the components of a sugar-specific Enzyme II complex within the PEP-PTS. This complex is responsible for the uptake and phosphorylation of specific sugars as they are transported into the bacterial cell. CelR directly regulates the expression of the ptcA, ptcB, and ptcC genes. Additionally, arcC (carbamate kinase) within the arginine deiminase system is involved in all experimental conditions; it is known for energy production and pH regulation in bacteria, especially in the acidic environment of the oral cavity. The gene bgl (putative phospho-beta-glucosidase) is likely involved in the metabolism of phosphorylated beta-glucosides, breaking them down into glucose-6-phosphate, which can then be used for energy production through glycolysis. This enzyme contributes to the ability of bacteria to utilize a wide range of carbohydrates, thereby supporting their growth, acid production, and overall virulence in the oral cavity [62] (right panel of Figure SM6).

Looking at the annotations of the genes identified across scorecards, frequently appearing keywords were the “PTS” (phosphotransferase) system (lacE, lacF, SMU_311, SMU_312, bglP, SMU_313, ptcA, ptcB, ptcC), cel (celR) or sorbitol (SMU_310, SMU_309) “operon”, and “acetyltransferase” (pdhC, lacA, pfl2, pdhC).

### Using the Scorecard on Dataset 4

The dataset included data on the transcriptome of the nosocomial pathogen *Enterococcus faecalis* (EF.peg.) when in contact with metal surfaces coated with AGXX^®^, an antimicrobial lining consisting of micro-galvanic elements formed by silver and ruthenium [63], Ag-coated V2A steel mesh, and uncoated V2A steel mesh. The transcriptomic data were analyzed through the scorecard to compare the genetic outcomes at the beginning or at the end of the experimental procedure (behavior of the three scaffolds at 90 minutes) as reported in Table SM6. Additionally, the scorecard analyses were carried out longitudinally, to evaluate differential expression over time (t=3, 6, 12, 24, 60, 90 minutes) throughout the experimental cycle.

#### Extreme Modifications Between Biomaterials at the Beginning or at the End of the Experimental Cycle

The heatmap in Figure SM7 contains the number of genes with RNA-sequencing expression falling inside the regions of interest of the standard scorecard. All the entries were in the first or third quadrants, indicating that the expression levels moved in the same direction; no entries were in regions **D** and **E**, implying extreme variation in one condition with no coexistent modifications in the other. At the beginning of the process, compared with V2A, Ag-coating resulted in more modifications (13 genes) than did AgXX^®^ vs. Ag and V2A (5 and 3 genes, respectively). At the end of the experimental procedure, the expression of a larger number of genes was modified; the scorecards involving AgXX^®^ vs. Ag and V2A identified 21 and 16 genes in quadrant I, respectively, and inside quadrant III, 16 and 15, respectively. On the Ag vs. V2A experiments after 90 minutes of exposure, the scorecard established a similar number of differentially expressed genes in quadrant I (24 genes) and quadrant III (18 genes, from the left panel of Figure SM7).

Table 4, whose rows were produced by one of the scorecard library’s supplementary functions, orders all genes regarding differential expression between experimental conditions. The largest difference was caused by Ag-coating and involved gene EF.peg.939 (ribosomal RNA large subunit methyltransferase N); methylation of nucleotides in ribosomal RNA could happen in all living organisms, but for the target bacteria, modifications were recently elucidated in [64]. Gene EF.peg.841 (no annotation available) was ubiquitously active both at the beginning and at the end of the laboratory experiments for V2A vs. Ag and AgXX^®^ vs. Ag-coating at the end of the tests only. The entry EF.peg.330 (dihydrolipoamide acyltransferase component of branched-chain alpha-keto acid dehydrogenase complex) was specifically active in experiments involving AgXX^®^ versus other materials after 90 minutes of bacteria exposure in the laboratory cycle. In *Enterococcus faecalis*, the enzyme dihydrolipoamide acyltransferase, also known as E2 component, within the branched-chain alpha-keto acid dehydrogenase complex, serves as a source of essential metabolic intermediates and energy in bacteria that thrive in nutrient-limited environments [65]. Among the several genes with large differences encountered for AgXX^®^, the symbol EF.peg.703 (PTS system2C mannose-specific IIA component) is part of the phosphoenolpyruvate-dependent sugar phosphotransferase system (PTS), responsible for the uptake and phosphorylation of sugars during transport across the bacterial membrane, regulating sugar uptake, energy generation, and even virulence because it is associated with the ability of *E. faecalis* to colonize human environments [66]. Linked to EF.peg.703, are EF.peg.702 (PTS system2C mannose-specific IIB component) and EF.peg.701 (PTS system2C mannose-specific IIC component), whereas EF.peg.540 (LSU ribosomal protein L25p) and EF.peg.540 (LSU ribosomal protein L25p) contributes to ribosome assembly by preserving the integrity of the ribosome’s active sites, including the peptidyl transferase center. The latter bioactivity, survival and adaptation to different environments, is also linked to EF.peg.949 (NAD-dependent malic enzyme), an enzyme effective for the interconversion of malate to pyruvate for energy production and carbon balance; hypothetically, inhibiting this enzyme may disrupt the energy production and metabolic flexibility of *E. faecalis*, reducing its ability to survive in hostile environments like the human host or biofilms.

**Table 4:** Top thirty largest expression differences between experimental conditions at the beginning (t03, t=3 minutes) or at the end (t90, t=90 minutes) of the laboratory tests on dataset 4

Cond. X	Cond. Y	Quadr.	ROI	Entry	Difference
V2A t=90min	Ag t=90min	Q3	B	EF.peg.939	8.1
V2A t=90min	Ag t=90min	Q3	A	EF.peg.841	4.87
AgXX t=90min	Ag t=90min	Q3	A	EF.peg.841	4.81
V2A t=3min	Ag t=3min	Q3	A	EF.peg.841	4.36
AgXX t=90min	Ag t=90min	Q1	B	EF.peg.330	4.24
AgXX t=90min	V2A t=90min	Q1	B	EF.peg.330	4.19
AgXX t=90min	V2A t=90min	Q1	C	EF.peg.951	3.94
AgXX t=90min	Ag t=90min	Q1	B	EF.peg.2991	3.49
AgXX t=3min	Ag t=3min	Q3	A	EF.peg.481	3.21
AgXX t=3min	V2A t=3min	Q3	A	EF.peg.481	3.21
AgXX t=90min	Ag t=90min	Q1	B	EF.peg.703	2.87
V2A t=90min	Ag t=90min	Q1	C	EF.peg.807	2.64
V2A t=90min	Ag t=90min	Q1	A	EF.peg.2991	2.53
AgXX t=90min	V2A t=90min	Q1	C	EF.peg.949	2.49
AgXX t=90min	Ag t=90min	Q1	A	EF.peg.702	2.37
AgXX t=90min	Ag t=90min	Q1	A	EF.peg.540	2.3
AgXX t=90min	Ag t=90min	Q1	B	EF.peg.1970	2.27
V2A t=90min	Ag t=90min	Q3	B	EF.peg.364	2.21
AgXX t=90min	V2A t=90min	Q1	A	EF.peg.540	2.19
AgXX t=90min	Ag t=90min	Q3	B	EF.peg.2355	2.14
AgXX t=90min	Ag t=90min	Q1	A	EF.peg.3041	2.05
AgXX t=90min	Ag t=90min	Q1	A	EF.peg.629	2.05
V2A t=90min	Ag t=90min	Q1	B	EF.peg.3044	2.03
AgXX t=90min	V2A t=90min	Q1	B	EF.peg.1970	1.95
AgXX t=90min	Ag t=90min	Q1	A	EF.peg.697	1.94
AgXX t=90min	Ag t=90min	Q1	B	EF.peg.696	1.93
V2A t=90min	Ag t=90min	Q1	B	EF.peg.55	1.92
AgXX t=90min	Ag t=90min	Q1	A	EF.peg.2374	1.9
AgXX t=90min	Ag t=90min	Q1	A	EF.peg.701	1.89
AgXX t=90min	Ag t=90min	Q1	A	EF.peg.698	1.87

Considering that the genes extremely expressed in the majority of the conditions, are summarized on the right side of Figure SM7, the scorecards revealed that genes identified as EF.peg.1868 and EF.peg.426 were active in all initial comparisons after 3 minutes, but their expression levels remained stable only for Ag vs. V2A at the end of the experiment. The exact initial behavior is observed for EF.peg.481, but it later maintains a high expression level exclusively for V2A vs. AgXX^®^. Gene EF.peg.992 was initially overexpressed only in Ag vs. V2A, and, after 90 minutes, it was found to be highly upregulated in all comparisons.

The evaluation of single genes active in only one experimental comparison is reported in Table SM3. At the beginning of the experimental procedure, the scorecard detected a few specific gene activations. In contrast, at the end of the experiment, peculiar subsets of genes could be identified for each comparison. Compared with the Ag-coating, the AgXX^®^ biomaterial explicitly activated EF.peg.3040 (copper-translocating P-type ATPase), and EF.peg.3041 (negative transcriptional regulator-copper transport operon) is in line with the hypotheses of [31] concerning the cop operon. Additionally, the AgXX^®^ vs. V2A analysis revealed the peculiar activation of EF.peg.951 (malate Na+ symporter), EF.peg.2473 (pneumococcal vaccine antigen A homolog) inside the first quadrant, and EF.peg.111 (site-specific recombinase2C phage integrase family) in the third quadrant. The malate Na+ symporter is a specific type of 2-hydroxycarboxylate transporter family member that mediates the symport of malate (a 2-hydroxycarboxylate) with sodium ions (Na+) across the cell membrane, which have been recognized also in *Enterococcus faecalis* [67]. Distinctive genetic symbols, such as EF.peg.1851 (putative transposon excisionase 3B Tn916 ORF1-like), could be found when comparing Ag-coated V2A to V2A both at the beginning of the experimental process and at the end. After 90 minutes of exposure to bacteria, the scorecard reported a peculiar difference in Ag-coated V2A vs V2A genes EF.peg.327, EF.peg.328, and EF.peg.329 (branched-chain alpha-keto acid dehydrogenase 2 C E1 component 2 C subunits), EF.peg.325 (butyrate kinase), EF.peg.324 (phosphate butyryltransferase), EF.peg.326 (dihydrolipoamide dehydrogenase of branched-chain alpha-keto acid dehydrogenase). Moreover, genes EF.peg.939 (ribosomal RNA large subunit methyltransferase N), EF.peg.3034 (Cystathionine beta-synthase), and EF.peg.1795 (Transposase IS3/IS911) were detected. The genes associated with symbols between EF.peg.324 and EF.peg.329 were all enzymes (or subunits) involved in metabolic pathways related to the catabolism of branched-chain amino acids and the production of short-chain fatty acids like butyrate.

#### Biomaterial–Specific Modifications Over Time

Figure SM8 shows the scorecards whose axes report the initial (t=3 minutes) and the final (t=90 minutes) sessions of the experiments for each biomaterial. For all three biomaterials, the variations moved in the same direction (over-expression in quadrant I, or underexpression in quadrant III): no genes displayed opposite behavior over time (quadrants II and IV). The material displaying the major changes over time was V2A, with 18 genes markedly underexpressed and 3 upregulated (Table 5).

For example, in Ag-coated metal, considering the genes not previously described, EF.peg.2384 (beta-galactosidase 3) was constantly underexpressed both at t=3 and t=90 minutes, together with the other not annotated genes (EF.peg.1868, EF.peg.2124, EF.peg.841, EF.peg.682, EF.peg.426, EF.peg.2437). During the changes over time in the experiments at t=12, 24, and 60 compared with 90 minutes (Table 5), the extremely modified genes for the Ag-coated samples were EF.peg.2174 (carbamate kinase) and EF.peg.2180 (beta-hexosaminidase). The carbamate kinase enzyme is involved primarily in the arginine deiminase (ADI) pathway, whereas EF.peg.2180 is related to the degradation of complex carbohydrates, specifically by cleaving N-acetylglucosamine (GlcNAc) residues from glycoproteins, glycolipids, and glycosaminoglycans. In AgXX^®^ at t=3 compared with t=90 minutes, there is a constant underexpression of EF.peg.2124 (hypothetical protein), other than EF.peg.3040 and EF.peg.3041 previously mentioned, and an overexpression that increases over time of the gene EF.peg.3039 (copper chaperone), which is responsible for safely transporting and delivering copper ions within cells to specific target proteins and cellular compartments where copper is needed. Considering the antimicrobial properties of copper, bacteria might rely on chaperone systems to defend themselves against Cu-mediated protein aggregation [68]. Additionally, genes EF.peg.92 and EF.peg.481 displayed over- and underregulation that increased over time. For the same biomaterial, genes whose expression changed over time at t=6, 12, 24, and 60 min. vs. 90 minutes (Table 5) included EF.peg.377 (CsbD-like), EF.peg.2991, and EF.peg.3039 (Copper chaperone). For V2A at t=3 vs. t=90 minutes, the scorecard revealed more extremely modified entries; among them, EF.peg.2456 (pyrroline-5-carboxylate reductase), and EF.peg.339 (membrane-bound protease 2 C) displayed minimal changes. Pyrroline-5-carboxylate reductase is an enzyme that plays a role in the biosynthesis of the amino acid proline from glutamate: proline is important for protein synthesis, particularly in conditions where cells are exposed to osmotic stress or oxidative stress [69], and in the context of human physiology, it plays a role in collagen synthesis and other metabolic processes. Considering shifts between scorecards’ areas of interest (reflecting consistent modifications over time of the expression levels) at t=12, 24, 60, compared with 90 minutes (Table 5), the gene EF.peg.124 (secreted endo-beta-N-acetylglucosaminidase), and EF.peg.383 appeared markedly upregulated. The enzyme encoded by the gene EF.peg.124 hydrolyzes the N-glycosidic bond in glycoproteins, specifically targeting the $\beta$−1,4 linkages in the N-acetylglucosamine (GlcNAc) chains of N-linked glycoproteins; by degrading glycoproteins found in the host, the bacteria can interfere with host immune responses [70]. Modifying host glycoproteins, endo-beta-N-acetylglucosaminidase could influence the composition and stability of biofilms, affecting persistence and resistance of *E. faecalis* in infections.

With respect to gene variation over time, in the case of V2A, two genes EF.peg.382 and EF.peg.383 were present in five scorecards at different time points, together with EF.peg.992. Genes EF.peg.382 (multiple sugar ABC transporter 2 C membrane-spanning permease protein MsmG) and EF.peg.383 (multiple sugar ABC transporter 2 C substrate-binding protein) was highly expressed in the first quadrant. The high expression observed in *Enterococcus faecalis* might indicate that the bacteria successfully survived on V2A longer than the Ag-coated or AgXX^®^ bacteria did. Using the scorecards over time, one could identify shifts between ROIs to track changes during the experimental cycle. For AgXX^®^, genes EF.peg.3039 (copper chaperone), EF.peg.3040 (copper-translocating P-type ATPase), and EF.peg.3041 (negative transcriptional regulator-copper transport) were identified by the scorecard throughout all five time points of the experiment. For example, in AgXX^®^, EF.peg.3039 (copper chaperone) is mildly expressed only at t=3 minutes, but at t=6 minutes it is already extremely overexpressed, suggesting early activation. In Ag-coated metal scaffolds and V2A, over time, genes EF.peg.992, EF.peg.1868, EF.peg.426, EF.peg.2491, EF.peg.383 appeared repeatedly over the scorecards.

**Table 5:** Number of extremely varying genes over time on each biomaterial according to the full scorecard (dataset 4)

X-axis	Y-axis	Q1 A	Q1 B	Q1 C	Q1 M	Q2 A	Q3 A	Q3 B	Q3 C	Q3 M
Ag t=90	Ag t=03	2	0	0	0	0	7	0	0	0
	Ag t=06	2	0	0	2	0	11	0	0	0
	Ag t=12	5	6	3	2	0	6	0	1	0
	Ag t=24	24	17	7	20	2	12	3	1	2
	Ag t=60	32	19	7	12	0	16	3	0	2
AgXX t=90	AgXX t=03	3	1	0	0	0	2	0	0	0
	AgXX t=06	4	1	1	1	0	5	0	0	0
	AgXX t=12	19	5	2	12	0	15	0	1	1
	AgXX t=24	40	2	12	21	0	11	1	2	1
	AgXX t=60	11	7	9	3	0	15	2	2	1
V2A t=90	V2A t=03	2	0	1	1	0	18	0	0	0
	V2A t=06	2	1	2	0	0	9	0	0	0
	V2A t=12	21	6	9	18	0	15	2	0	1
	V2A t=24	35	10	17	41	0	22	13	1	15
	V2A t=60	15	12	13	15	0	18	12	0	6

## Discussion

 A Python library has been released to narrow down transcriptomic data and extract the most relevant information arising from expression values when confronting multiple experimental conditions. The scorecard displays extreme variations selected during pairwise comparisons based on $log_{2}$ fold-change thresholds and statistical significance, reporting them on a Cartesian plane that summarizes all possible expression level outcomes. The software enhances the standard four-way plot found in an R library and includes additional analysis capabilities. This software tool enables a comprehensive comparison by facilitating the identification of genes with consistent or divergent regulation patterns across multiple treatments or time points, and by allowing the detection of genes with condition-specific expression changes, thereby supporting the understanding of relevant biological processes. In addition, the scorecard helps reduce the amount of data by summarizing complex, high-dimensional data in a more manageable and interpretable format, providing a clear visual summary that aids in communicating results to a broader audience, including those who may not be experts in OMICS data interpretation.  Using this scatterplot, one can identify genes with unexpected expression patterns that warrant further investigation, therefore facilitating the detection of outliers or anomalous data points that may indicate interesting biological phenomena or potential technical issues. Regarding the interpretation of the results, the scorecard facilitates the distinction between global and specific gene expression responses, providing a context for understanding how gene expression changes in response to multiple factors or treatments simultaneously. Another advantage is that it supports exploratory data analysis, leading to new insights and directions for future research or generating new hypotheses about gene regulation and interaction networks based on observed expression patterns. Moreover, it confirms whether observed trends are consistent across multiple conditions, increasing confidence in the results.

With respect to the analysis of dataset 1, several genes were extremely expressed when bacteria were exposed to vancomycin, a peptide antibiotic, that has long been a mainstay in the treatment of serious gram-positive bacterial infections, particularly those caused by *Staphylococcus aureus* [71]. The drug’s unique pharmacodynamic properties, such as its ability to maintain high and prolonged blood levels, its bactericidal action, and the lack of development of resistant strains, contribute to its clinical significance, especially in instances where other antibiotics may be less effective [72]. Previous studies have underscored its effectiveness against methicillin-resistant strains, establishing it as a critical option for managing these challenging infections [73]. However, the increasing prevalence of strains exhibiting reduced susceptibility to glycopeptides has raised concerns about the long-term efficacy of vancomycin [74]. Vancomycin displays distinct characteristics compared with other antibiotics used for *Staphylococcus aureus* infections, such as linezolid, ceftaroline, and delafloxacin. *Staphylococcus aureus* can develop resistance to vancomycin through the acquisition of specific genes, typically referred to as van genes (e.g., vanA, vanB), which alter the target of the antibiotic [75]. While linezolid offers the benefit of oral administration and exhibits activity against both gram-positive and certain gram-negative bacteria, it may have limitations in treating severe infections because of its bacteriostatic nature, which can be less effective in critically ill patients than vancomycin’s bactericidal properties [72]. The extremely bioactive protein for VAN encoded by SAUSA300_RS03455 (auxiliary protein GraX/ApsX), a two-component regulatory system that helps *Staphylococcus aureus* sense and respond to cell envelope stress, particularly from CAMPs and other environmental challenges. *Staphylococcus aureus* cells under vancomycin pressure often experience cell wall stress. Additionally, ceftaroline presents broad-spectrum activity, particularly against methicillin-resistant *Staphylococcus aureus*, yet may not demonstrate the same level of tolerability in certain high-risk populations, highlighting the need for individualized treatment approaches based on patient-specific factors [76–78]. The scorecard found SAUSA300_RS08475 (shikimate dehydrogenase) linked to CPT: it is an enzyme involved in the shikimate pathway, which is crucial for the biosynthesis of aromatic amino acids (such as phenylalanine, tyrosine, and tryptophan) in bacteria, fungi, plants, and some protozoans; the shikimate pathway is not present in mammals, making it an attractive target for antibiotics and herbicides. While shikimate dehydrogenase is not directly related to the mechanism of action of ceftaroline, it is vital for the survival and growth of *Staphylococcus aureus* [79]. Compared with other antibiotics, delafloxacin’s unique dual mechanism of action targets both gram-positive and gram-negative bacteria; nonetheless, concerns regarding its place in therapy, especially in comparison to established agents such as vancomycin, remain, particularly in terms of clinical response and safety considerations [80]. The scorecard revealed that SAUSA300_RS03525 (DUF402 domain-containing protein) and SAUSA300_RS04560 (NAD(P)/FAD-dependent oxidoreductase) were markedly overexpressed in CPT, DEL, and LZD.

For dataset 2, the scorecard considered numerous experimental conditions involving methicillin-resistant *Staphylococcus aureus* to evaluate how adjuvants could potentiate antibiotics for biofilm disruption. In [81, 82], research has shown that loratadine, an FDA-approved antihistamine, effectively enhances the activity of cell wall-active antibiotics against various strains of MRSA. Additionally, loratadine and oxacillin have successfully disrupted preformed biofilms and prevented their formation in vitro. A structurally similar compound, brominated carbazole known as compound 8, has also demonstrated antibiotic potentiation [83, 84]. When considered alone, Ox massively positively activated the expression of SA_RS03110 (C1q-binding complement inhibitor VraX), SA_RS13430 (cell wall inhibition responsive protein CwrA), and SA_RS08315 (DUF4930 family protein). Lor follows the same positive bioactivation, whereas Compd8 has mild negative expression; theoretically, under the view of potentiating Ox action, Lor might enhance bioactivation, while Compd8, which displayed opposite behavior to oxacillin, might support countering the bacterium’s immune evasion tactics, not directly involved in the mechanism of resistance to oxacillin. VraX is a protein in *Staphylococcus aureus* that has been identified as a C1q-binding complement inhibitor: it is associated with the bacterium’s ability to evade the host immune system, particularly the complement system, which is a key part of innate immunity [44]. The DUF4930 proteins (domain of unknown function 4930) [85] are a membrane-related, and given that oxacillin is a $\beta$-lactam antibiotic targeting cell wall synthesis by binding to penicillin-binding proteins, any protein associated with cell wall metabolism, modification, or regulation could indirectly influence the effectiveness of oxacillin. Similar considerations could be made for SA_RS13430, with Lor following the same activation pattern as Ox (left side of Figure SM3). The SA_RS03110 gene is exceptionally active in 63 comparisons over 105, as summarized on the right side of Figure SM3. In contrast, genes extremely active in only one experimental condition are included in Table 2: exploring this table, one could look for experiment-specific genetic signatures. In Ox and Compd8 compared with Lor, genes found positively bioactivated were SA_RS12530 (nitrate reductase subunit beta), SA_RS12545 (nitrite reductase small subunit NirD), and SA_RS12550 (nitrite reductase large subunit NirB). Compared to Ox alone, the addition of Compd8 and Lor to Ox resulted in the specific activation of SA_RS05690 (complement convertase inhibitor Efb), SA_RS05695 (complement inhibitor SCIN-B), SA_RS01280 (pyruvate formate-lyase-activating protein), and SA_RS03250 (alcohol dehydrogenase AdhP). More negatively expressed for Compd8 co-treatment (fold-change −3.95) than Lor co-treatment (−1.98), SA_RS05695 helps the bacterium evade the host immune system by inhibiting the complement system, specifically the C3 convertase enzyme complex: the C3 convertase is essential for the activation of the complement cascade, leading to opsonization (marking pathogens for phagocytosis), the recruitment of immune cells, and the formation of the membrane attack complex (MAC), which can lyse bacteria [86]. A minor difference was observed for SA_RS05690, while SA_RS01280 was markedly underexpressed in response to Lor cotreatment and did not significantly change in response to Compd8 cotreatment. As shown in the bar plots in Figure SM2, the genes SA_RS09375 (peptidylprolyl isomerase) and SA_RS09765 (cell wall-active antibiotic response protein LiaF) were markedly overexpressed following Lor_Ox cotreatment compared with the Cmpd8_Ox adjunction. Both of these genes can be considered essential for bacterial survival: the first maintains the bacterium's proteins in a functional state. It protects against misfolding, which could otherwise lead to cell death, thereby helping bacteria survive in harsh conditions. The latter is connected to *staphylococcus aureus*’s LiaF protein, assisting the bacterium in reacting to antibiotics that act on the cell wall. LiaF is a component of the three-component LiaFSR (Lipid II-interacting Antibiotic sensor-regulator) system, which aids bacteria in identifying and defending against the effects of antibiotics that attack their cell walls [87].

Bacteria within the biofilm metabolize fermentable carbohydrates, producing lactic acid and other acids: *Streptococcus mutans* is particularly adept at thriving in acidic environments and continuing acid production even when the pH decreases. The acids lower the pH, affecting the environment of the tooth surface: a pH below 5.5 is critical because it demineralizes tooth enamel, which is the first step in forming cavities [88]. In [29], lactose and xylitol were particularly effective in modulating IPS metabolism, maintaining cell wall integrity, and influencing overall virulence during the early phases of biofilm formation and structure. However, they exhibited an inverse relationship compared with sucrose and glucose. The scorecard analysis of dataset 3, revealed that a highly bioactive gene was SMU_494, always downregulated for Suc. The pdhC gene (possibly the acetoin dehydrogenase E2 component) followed the same pattern of SMU_494 across the experiments. This gene encodes one of the subunits of the pyruvate dehydrogenase complex (PDH complex), specifically the dihydrolipoamide acetyltransferase component (E2), which catalyzes the conversion of pyruvate, a key product of glycolysis, into acetyl-CoA for cellular metabolism, linking glycolysis to the citric acid cycle [89]. Another gene that follows the same bioactivation pattern encountered for pdhC and SMU_494 was treA (putative trehalose-6-phosphate hydrolase), which is essential for the metabolism of trehalose [90]. In the oral cavity, *S. mutans* is exposed to fluctuating nutrient levels: the ability to metabolize various sugars, including trehalose (a disaccharide composed of two glucose molecules), gives the bacterium metabolic flexibility, allowing it to thrive in different environments (for example, during pH fluctuations), such as when sucrose is less available. Genes SMU_313 (putative PTS system) and SMU_314 (unknown annotation but reported in [91] with PTS involvement) were highly expressed under five conditions: the phosphoenolpyruvate-phosphotransferase system (PEP-PTS) couples the transport of sugars across the bacterial cell membrane with their phosphorylation [92]. Among the components of PEP-PTS are enzyme II (EII), a membrane-bound protein complex specific to each sugar; it has multiple domains (IIA, IIB, IIC) that work together to both transport the sugar into the cell and phosphorylate it. Indeed, scorecard analysis detected marked expression of the previously described ptcA (involved in the initial transfer of the phosphate group from HPr to the sugar-specific complex), ptcB (encoding the IIB component, which is responsible for transferring the phosphate group to the sugar itself as it is transported across the membrane), ptcC (encoding the IIC component, which forms the membrane-spanning channel through which the sugar is transported into the cell), bgl, and celR (directly regulating the expression of the ptcA, ptcB, and ptcC genes). Notably, another gene pttB, frequently occurring across scorecards, is associated with the same PTS mechanism. The scorecard entries also included genes connected to carbon catabolite repression (CCR) which has another role: CCR ensures that the cell’s resources are directed towards the most energy-efficient and easily metabolizable carbon sources, such as glucose, thus optimizing growth and survival in environments with multiple potential nutrient sources, whereas PEP-PTS is a sugar transport system that couples the uptake of specific sugars with their phosphorylation.

In dataset 4, the work of [31] reported that exposure to the AgXX^®^ coating significantly affected the gene expression of *E. faecalis*, with a large effect on the transcriptome and the upregulation of environmental stress-related genes. These findings suggest that AgXX^®^ induces a broad general stress response in *E. faecalis*, potentially through a mechanism involving the combination of Ag+ ions and reactive oxygen species. Indeed, the authors theorized that the creation of reactive oxygen species such as hydroxyl radicals is related to the bioavailability of free copper ions mediating antimicrobial action; about this biological process, the scorecard highlighted the extreme bioactivation of EF.peg.3040 (copper-translocating P-type ATPase) and EF.peg.3041 (negative transcriptional regulator-copper transport operon) on AgXX^®^ compared with the silver coating. Other genes also constituted specific signatures of Ag. vs AgXX^®^ comparison after 90 minutes of biofilm formation, but the annotation was unavailable thus reducing the interpretation of the findings. According to scorecard results comparing genes activated across biomaterials, the genetic signatures connected to specific biomaterials were EF.peg.1868 and EF.peg.426 with sustained overexpression for Ag-coating and V2A in the final stages of biofilm exposure, and EF.peg.481 for Ag-coating vs. AgXX^®^. Additionally, entry EF.peg.2124 maintained its downregulation over time only for Ag vs. AgXX^®^, whereas EF.peg.2384 (beta-galactosidase 3) remained underregulated for Ag vs. V2A only. This latter enzyme is responsible for breaking down beta-galactosides into monosaccharides, typically glucose and galactose, which can then be used as sources of energy and carbon [93]. Considering changes over time for each biomaterial, modifications were mainly sustained in specific genes without noticeable shifts between quadrants or scorecard regions.

## Conclusions

Four biofilm-related datasets were analyzed through a novel free software library written in Python that tracks fold changes and statistical significance, facilitating the evaluation of multiple comparisons between experimental conditions. The software generates a scorecard to evaluate extreme or moderate expression level modifications quickly and prepares additional files to support the analysis. The software library could aid bioinformatics or data scientists interested in easing and automating the analyses of datasets under manifold experimental conditions.

On dataset 1, when VAN was involved, the scorecard detected extremely expressed genes related to CAMP resistance, protein dephosphorylation, cell wall maintenance, and biofilm formation. For other antibiotics, biological processes also regulate virulence through quorum sensing, biofilm formation, nucleic acid production, or efflux of drugs and toxic compounds, enhancing bacteria’s resilience. On dataset 2, when considering all experimental comparisons, the scorecard analysis identified 1126 genes falling within the outer regions of interest. Frequently, the highly active genes were related to maintaining cell wall integrity, nitrate reductase process for respiration in oxygen-poor environments (allowing the bacteria to continue to replicate, produce toxins, and dodge the immune system eventually contributing to biofilm stability in anaerobic regions), evasion of the host immune system also triggering proinflammatory response, reducing opsonization and preventing phagocytosis, lysis of leukocytes (aiding bacterial invasion) and erythrocytes (to acquire iron for bacterial growth). On dataset 3, the biological processes mainly involved aspects of carbohydrate uptake and metabolism through the phosphotransferase system. In contrast, in dataset 4, several genes were associated with energy production, carbon metabolism, the PTS, or metal-ion processing.

## Supplementary Information

Below is the link to the electronic supplementary material.

Supplementary file 1 (pdf 4444 KB)

## Data Availability

$\bullet$ Software name: A scorecard to compare wet-lab experimental conditions $\bullet$ Software home page: https://github.com/m89p067/Scorecard $\bullet$ Archived version: 10.5281/zenodo.13808354 $\bullet$ Operating system(s): Platform independent $\bullet$ Programming language: Python 3 $\bullet$ Other requirements: adjustText 1.2.0, matplotlib 3.9.1, numpy 2.0.1, pandas 2.2.2, scipy 1.14.0, statsmodels 0.14.2 $\bullet$ License: GPL-3.0 license

## Abbreviations

Ag: Ssilver-coating (in dataset 4 analysis)
AIP: Autoinducing peptide
CAMP: Cationic antimicrobial peptide
CCR: Carbon catabolite repression
Cmpd8: Ccompound 8 (in dataset 2 analysis)
CPT: Ceftaroline (in dataset 1 analysis)
CSV: Comma-separated values (file format)
DE: Differential expression
DEL: Delafloxacin (in dataset 1 analysis)
DUF: Domain of unknown function
FAD: Flavin adenine dinucleotide
FPKM: Fragments per kilobase of transcript per million mapped reads
Glu: Glucose (in dataset 3 analysis)
JSON: JavaScript object notation (file format)
Lac: Lactose
Lor: Loratadine (in dataset 2 analysis)
LZD: Linezolid (in dataset 1 analysis)
MRSA: Methicillin-resistant *Staphylococcus aureus*
NAD: Nicotinamide adenine dinucleotide
ORF: Ddifferential open reading frame (in dataset 1 analysis)
Ox: Oxacillin (in dataset 2 analysis)
PBP: Penicillin-binding protein
PTS: Phosphotransferase system
Q1: (scorecard) Quadrant I
Q2: (scorecard) Quadrant II
Q3: (scorecard) Quadrant III
Q4: (scorecard) Quadrant IV
RNA-seq: RNA sequencing
ROI: (scorecard) Region of interest
SA: *Staphylococcus aureus* USA 100 (in dataset 2 analysis)
SAUSA300: *Staphylococcus aureus* USA 300 (in dataset 1 analysis)
SMU: *Streptococcus mutans*
Suc: Sucrose (in dataset 3 analysis)
STK: Serine–threonine kinases
V2A: stainless-steel mesh (in dataset 4 analysis)
VAN: Vancomycin (in dataset 1 analysis)
Un: Untreated
