# Supplementary material for: A Scorecard for Information Synthesis in Multiple Experimental Conditions: Application to Bacterial Biofilm Matrix Transcriptomics
Source: Curr Microbiol. 2025 Sep 9;82(10):497. doi: 10.1007/s00284-025-04435-3 (PMC12420718; doi:10.1007/s00284-025-04435-3)
Supplement: Supplementary file 1 — (pdf 4444 KB) [file 284_2025_4435_MOESM1_ESM.pdf]

A scorecard for information synthesis in multiple experimental conditions: application to bacterial biofilm matrix transcriptomics

Mauro Nascimben[1] and Lia Rimondini[1]

[1]Department of Health Sciences, Università del Piemonte Orientale UPO, Corso Trieste 15/A, Novara, 28100, Italy.

SUPPLEMENTARY MATERIALS

Contents

1 Additional figures **2**

2 Additional tables **6**

3 Computational resources **10**

4 Dataset information **10**

5 Colors of the scorecards **13**

6 Scorecard software processing information **15**

7 A tutorial on a toy dataset **18**

1 Additional figures

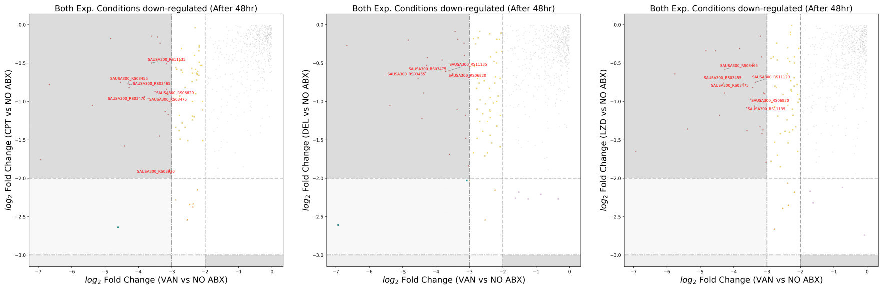

Fig. 1 The three Q3 values of the VAN vs. CPT, VAN vs. DEL, and VAN vs. LZD comparisons (dataset 1). All identified genes fall inside region of interest **D**.

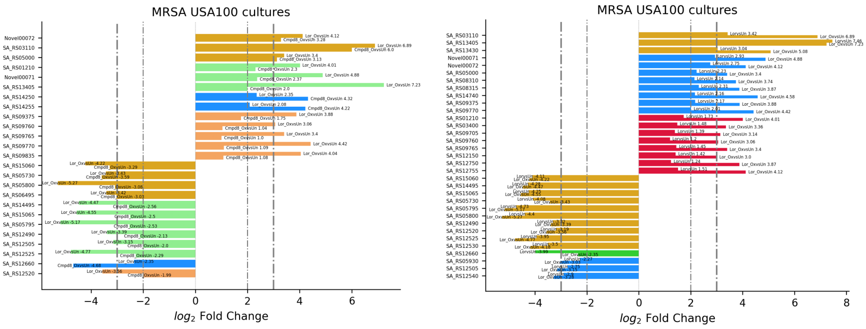

Fig. 2 On the left: Expression levels of the Lor_Ox cotreatment vs. untreated to Compd8_Ox cotreatment vs. untreated (dataset 2). On the right: Comparison between Lor vs. Un and Lor_Ox vs. Un (dataset 2).

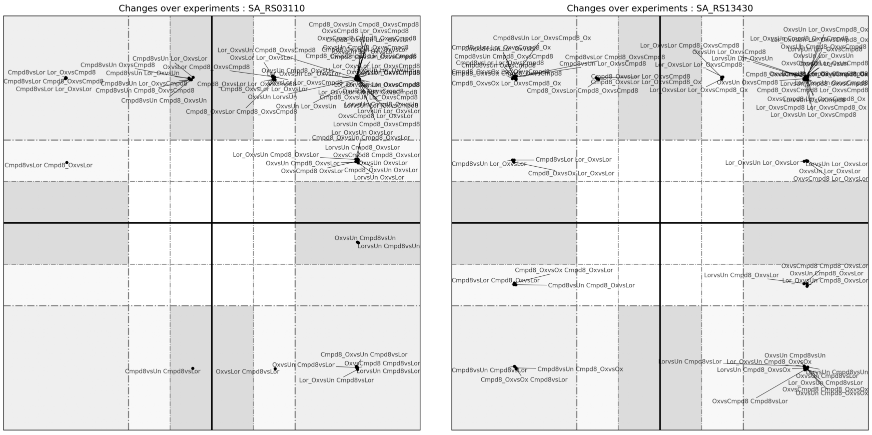

Fig. 3 Left image: occurrence of SA_RS13430 in different regions of the scorecard (dataset 2). This image has been created by consolidating the results of individual scorecards via a specialized function in the software library. The right image shows the positioning of SA_RS03110 when all possible combinations of experimental conditions are considered (dataset 2).

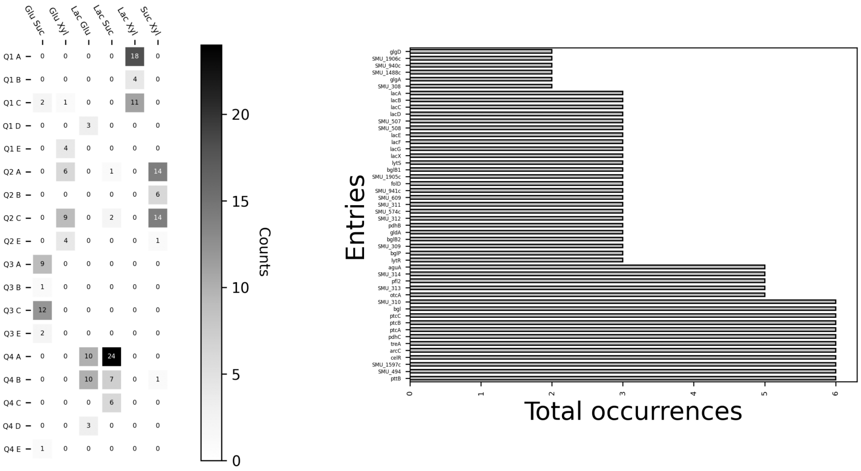

Fig. 4 On the left: Number of identified genes in dataset 3. On the right: Number of genes appearing on the scorecard ROIs across experiments (dataset 3).

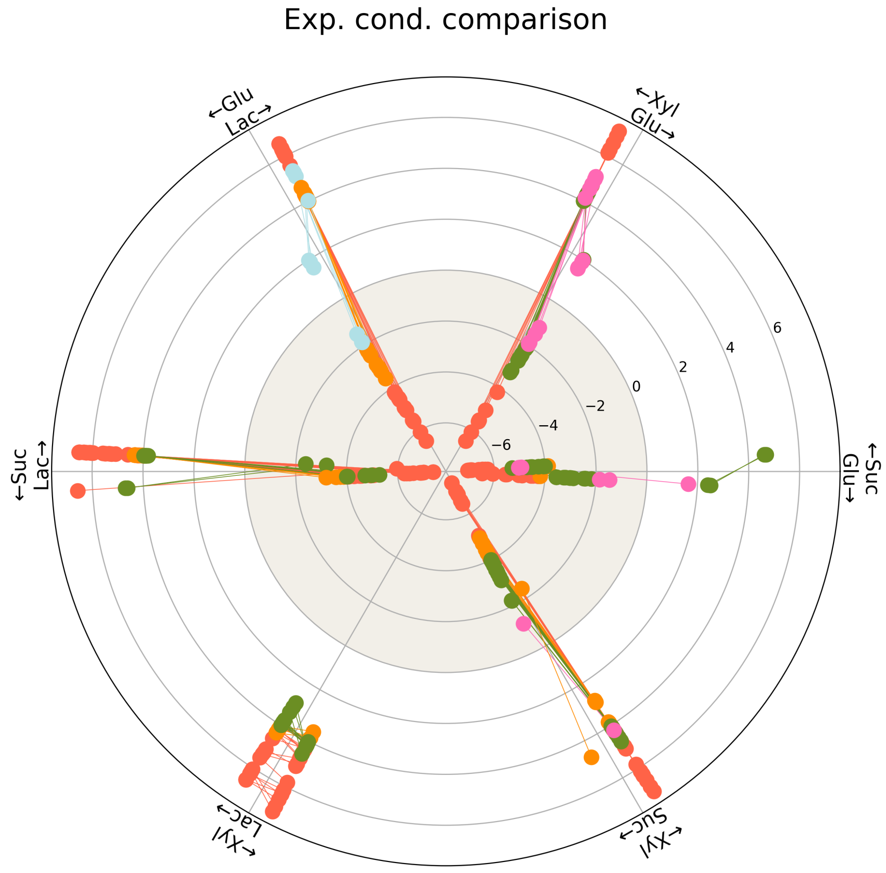

Fig. 5 Overview of all the comparison results on dataset 3 applying the standard scorecard.

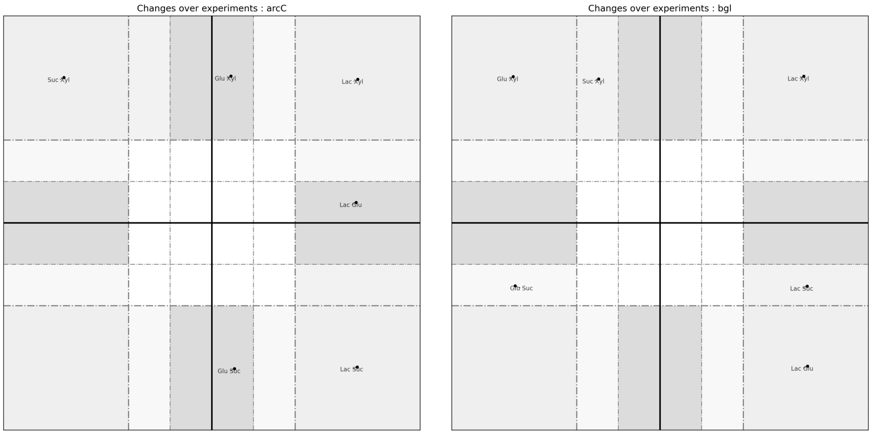

Fig. 6 Relative positioning of arcC (left image), and bgl (right image) on the scorecards throughout experiments (dataset 3): the involvement of the four sugars produced distinct activation patterns. These plots were created with an ad-hoc function that tracks the quadrants and regions of interest where a gene falls over time.

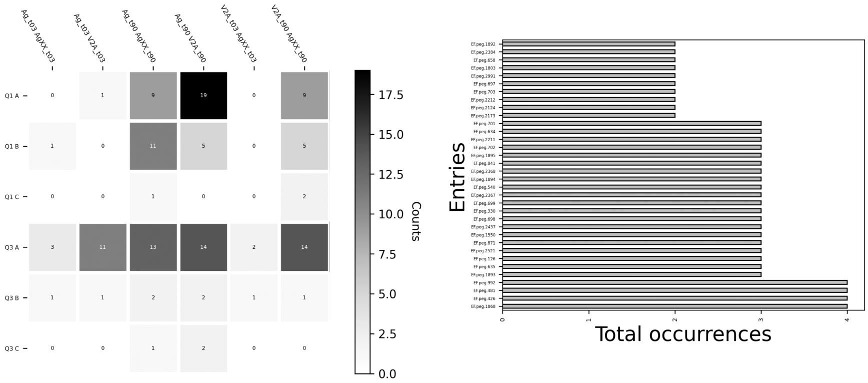

Fig. 7 On the left: Number of identified genes applying the scorecard to dataset 4. On the right: Common gene counts during dataset 4 analysis.

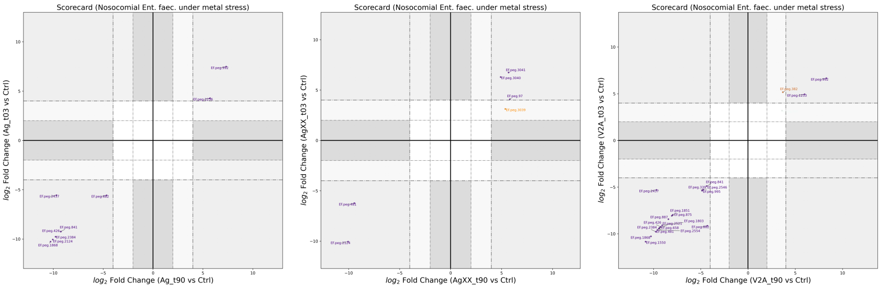

Fig. 8 The three standard scorecards for each biomaterial comparing extreme expression levels in the initial (t=3 min.) and final (t=90 min) stages of the research: on the left Ag-coated, in the center V2A, and in the right AgXX® (dataset 4).

2 Additional tables

Table 1 Experimental conditions where regions of the scorecard contained more than 10 entries (dataset 2).

X-axis	Y-axis	Q1 A	Q1 B	Q1 C	Q1 D	Q1 E	Q2 A	Q2 B	Q2 C	Q2 E	Q3 A	Q3 B	Q3 C	Q3 D	Q3 E	Q4 A	Q4 B	Q4 D
LorvsUn	Lor_OxvsOx	1	0	1	0	0	0	0	0	0	10	1	3	0	2	0	0	0
LorvsUn	Lor_OxvsUn	3	0	8	0	8	0	0	0	0	10	1	3	0	0	0	0	0
Lor_OxvsCmpd8	Lor_OxvsCmpd8_Ox	6	12	0	3	0	0	0	0	0	3	2	0	0	0	0	0	0
Lor_OxvsLor	Lor_OxvsCmpd8	2	0	8	0	11	0	0	0	0	0	0	0	0	0	0	0	0
Lor_OxvsOx	OxvsLor	0	0	0	0	0	11	2	4	0	0	0	0	0	0	1	1	0
Lor_OxvsUn	Lor_OxvsCmpd8	20	1	3	0	0	0	0	0	0	6	3	0	0	0	0	0	0
Lor_OxvsUn	Lor_OxvsCmpd8_Ox	6	11	0	1	0	0	0	0	0	0	1	0	2	0	0	0	0
Lor_OxvsUn	Lor_OxvsLor	2	7	0	10	0	0	0	0	0	0	0	0	0	0	0	0	0
Lor_OxvsUn	Lor_OxvsOx	1	2	1	2	0	0	0	0	0	11	2	5	0	0	0	0	0
Lor_OxvsUn	OxvsCmpd8	10	8	1	0	0	1	0	1	0	0	0	0	0	0	0	0	1
OxvsCmpd8	Cmpd8_OxvsOx	0	0	0	0	0	2	0	2	0	0	0	0	0	0	13	2	0
OxvsCmpd8	Lor_OxvsCmpd8	11	7	9	0	0	0	0	0	1	2	0	0	0	0	0	0	0

Note: Regions of interests without entries were excluded (all zeros columns).

Table 2 Regions of the scorecard fixing the second term of comparison (dataset 2).

X-axis	Y-axis	Q1 A	Q1 B	Q1 C	Q1 D	Q1 E	Q2 A	Q2 B	Q2 C	Q2 D	Q2 E	Q3 A	Q3 B	Q3 C	Q3 D	Q3 E	Q4 A	Q4 B	Q4 C	Q4 D
Cmpd8_OxvsCmpd8	Lor_OxvsCmpd8	5	0	3	0	4	0	0	0	0	0	0	0	0	0	0	0	0	0	0
OxvsCmpd8	Cmpd8_OxvsCmpd8	5	1	0	0	0	0	0	0	0	0	0	0	0	0	0	0	0	0	0
OxvsCmpd8	Lor_OxvsCmpd8	11	7	9	0	0	0	0	0	0	1	2	0	0	0	0	0	0	0	0
Cmpd8vsLor	Cmpd8_OxvsLor	2	0	0	0	0	0	1	0	0	0	2	1	0	1	0	0	0	0	0
Cmpd8vsLor	Lor_OxvsLor	0	0	0	0	0	1	1	0	4	0	0	0	0	0	0	0	0	0	0
Cmpd8_OxvsLor	Lor_OxvsLor	0	0	1	0	0	0	0	0	0	0	0	0	0	0	0	0	0	0	0
OxvsLor	Cmpd8vsLor	2	2	0	0	0	0	0	0	0	0	2	0	0	0	0	0	0	1	0
OxvsLor	Cmpd8_OxvsLor	0	0	0	0	0	0	0	0	0	0	2	0	0	0	0	0	0	0	0
OxvsLor	Lor_OxvsLor	0	0	1	0	1	0	0	0	0	0	0	0	0	0	0	0	0	0	0
Cmpd8_OxvsOx	Lor_OxvsOx	0	0	0	2	1	0	0	0	0	0	2	5	4	4	2	0	0	0	0
Cmpd8vsUn	Cmpd8_OxvsUn	2	0	2	0	0	0	0	0	0	1	4	4	0	0	0	0	0	0	0
Cmpd8vsUn	Lor_OxvsUn	0	0	0	0	1	1	0	2	0	3	0	1	0	0	0	0	0	0	0
Cmpd8_OxvsUn	Lor_OxvsUn	3	2	3	0	5	0	0	0	0	0	4	1	6	0	1	0	0	0	0
LorvsUn	Cmpd8vsUn	0	0	0	1	0	0	0	0	0	0	1	0	2	0	0	1	0	0	1
LorvsUn	Cmpd8_OxvsUn	1	1	2	1	1	0	0	0	0	0	5	5	0	1	0	0	0	0	0
LorvsUn	Lor_OxvsUn	3	0	8	0	8	0	0	0	0	0	10	1	3	0	0	0	0	0	0
OxvsUn	Cmpd8vsUn	0	0	0	0	0	0	0	0	0	0	0	0	0	0	0	1	2	0	1
OxvsUn	Cmpd8_OxvsUn	2	1	0	0	0	0	0	0	0	0	0	0	0	0	0	0	0	0	0
OxvsUn	LorvsUn	2	5	0	0	0	0	0	0	0	0	0	0	0	0	0	0	0	0	0
OxvsUn	Lor_OxvsUn	7	0	7	0	0	0	0	0	0	0	0	0	0	0	0	0	0	0	0

Note: Regions of interest without entries were excluded (all zeros columns).

Table 3 Genes identified by the scorecard bioactive in only one experimental condition (dataset 4).

X-axis	Y-axis	Quadr.	ROI	Symbol	Description
Ag t=3min	AgXX t=3min	Q1	B	EF.peg.402	FIG00631796: hypothetical protein
Ag t=3min	V2A t=3min	Q3	A	EF.peg.2404	hypothetical protein
Ag t=3min	V2A t=3min	Q3	A	EF.peg.2582	FIG00629850: hypothetical protein
Ag t=3min	V2A t=3min	Q3	A	EF.peg.3020	FIG00632450: hypothetical protein
Ag t=3min	V2A t=3min	Q3	A	EF.peg.875	hypothetical protein
Ag t=3min	V2A t=3min	Q3	A	EF.peg.1851	putative transposon excisionase 3B Tn916 ORF1-like
Ag t=90min	AgXX t=90min	Q1	B	EF.peg.3040	Copper-translocating P-type ATPase
Ag t=90min	AgXX t=90min	Q1	B	EF.peg.3041	Negative transcriptional regulator-copper transport operon
Ag t=90min	AgXX t=90min	Q3	A	EF.peg.620	hypothetical protein
Ag t=90min	AgXX t=90min	Q3	A	EF.peg.452	FIG00628160: hypothetical protein
Ag t=90min	AgXX t=90min	Q3	A	EF.peg.1458	hypothetical protein
Ag t=90min	AgXX t=90min	Q3	A	EF.peg.243	hypothetical protein
Ag t=90min	AgXX t=90min	Q3	A	EF.peg.1219	FIG00632207: hypothetical protein
Ag t=90min	AgXX t=90min	Q3	A	EF.peg.1074	hypothetical protein
Ag t=90min	AgXX t=90min	Q3	A	EF.peg.3070	FIG00630419: hypothetical protein
Ag t=90min	V2A t=90min	Q1	A	EF.peg.328	Branched-chain alpha-keto acid dehydrogenase2C E1 component2C alpha subunit
Ag t=90min	V2A t=90min	Q1	A	EF.peg.327	Branched-chain alpha-keto acid dehydrogenase2C E1 component2C alpha subunit
Ag t=90min	V2A t=90min	Q1	A	EF.peg.325	Butyrate kinase
Ag t=90min	V2A t=90min	Q1	A	EF.peg.324	Phosphate butyryltransferase
Ag t=90min	V2A t=90min	Q1	A	EF.peg.329	Branched-chain alpha-keto acid dehydrogenase 2C E1 component2C beta subunit
Ag t=90min	V2A t=90min	Q1	A	EF.peg.326	Dihydrolipoamide dehydrogenase of branched-chain alpha-keto acid dehydrogenase
Ag t=90min	V2A t=90min	Q3	A	EF.peg.8	FIG00632045: hypothetical protein
Ag t=90min	V2A t=90min	Q3	A	EF.peg.1795	Transposase IS3/IS911
Ag t=90min	V2A t=90min	Q3	A	EF.peg.886	FIG00629745: hypothetical protein
Ag t=90min	V2A t=90min	Q3	A	EF.peg.589	FIG00629715: hypothetical protein
Ag t=90min	V2A t=90min	Q3	A	EF.peg.2438	FIG00631881: hypothetical protein
Ag t=90min	V2A t=90min	Q3	B	EF.peg.939	Ribosomal RNA large subunit methyltransferase N
Ag t=90min	V2A t=90min	Q3	C	EF.peg.3034	Cystathionine beta-synthase
V2A t=90min	AgXX t=90min	Q1	B	EF.peg.809	FIG00629162: hypothetical protein
V2A t=90min	AgXX t=90min	Q1	C	EF.peg.951	Malate Na(+) symporter
V2A t=90min	AgXX t=90min	Q3	A	EF.peg.2473	Pneumococcal vaccine antigen A homolog
V2A t=90min	AgXX t=90min	Q3	A	EF.peg.111	site-specific recombinase2C phage integrase family
V2A t=90min	AgXX t=90min	Q3	A	EF.peg.2531	FIG00627385: hypothetical protein
V2A t=90min	AgXX t=90min	Q3	A	EF.peg.2554	FIG00630904: hypothetical protein
V2A t=90min	AgXX t=90min	Q3	A	EF.peg.887	FIG00633631: hypothetical protein

3 Computational resources

Every numerical experiment was accomplished on standard hardware, via a laptop computer with an Intel i5 CPU and 16 GB of RAM. Executing the scorecard and related functions did not require cloud or cluster computing resources, thus guaranteeing the reproducibility of the current analytical sequence by other researchers on standard computers.

4 Dataset information

The number of genes analysed through the scorecard in dataset 1 is reported in Table 4. The scorecards were produced by setting a fold change threshold of 2, a multiplication factor of 1.5 (consequently, the second fold-change threshold was 3), and a significance threshold of 0.05. For dataset 2, the number of genes analysed through the scorecard is included in Table 7. The scorecards were produced by setting a fold change threshold of 2, a multiplication factor of 1.5 (the second fold-change threshold was 3), and a significance threshold of 0.05. For dataset 3, the number of genes analyzed by the scorecard is included in Table 5. The scorecards were produced by setting a fold change threshold of 2, a multiplication factor of 2 (the second fold-change threshold was 4), and a significance threshold of 0.001. The analysis of dataset 4 employed a fold change threshold of 2.5, a multiplication factor of 2.5 (the second fold-change threshold was 5), and a significance threshold of 0.001.

Table 7: Dataset 2 information

X-axis	Y-axis	Tot. genes	Q1	Q2	Q3	Q4
Cmpd8vsLor	Cmpd8_OxvsCmpd8	3177	428	1036	505	1208
Cmpd8vsLor	Cmpd8_OxvsLor	3180	1141	491	1062	486
Cmpd8vsLor	Lor_OxvsCmpd8	3167	387	1170	361	1249
Cmpd8vsLor	Lor_OxvsCmpd8_Ox	3164	473	1067	473	1151
Cmpd8vsLor	Lor_OxvsLor	3164	684	871	679	930
Cmpd8vsUn	Cmpd8vsLor	3173	898	738	855	682
Cmpd8vsUn	Cmpd8_OxvsCmpd8	3173	696	764	829	884
Cmpd8vsUn	Cmpd8_OxvsLor	3172	888	744	854	686
Cmpd8vsUn	Cmpd8_OxvsOx	3170	1061	517	1082	510
Cmpd8vsUn	Cmpd8_OxvsUn	3183	1108	381	1229	465
Cmpd8vsUn	Lor_OxvsCmpd8	3167	711	846	741	869
Cmpd8vsUn	Lor_OxvsCmpd8_Ox	3162	709	831	762	860
Cmpd8vsUn	Lor_OxvsLor	3158	771	785	807	795
Cmpd8vsUn	Lor_OxvsOx	3155	903	641	952	659
Cmpd8vsUn	Lor_OxvsUn	3168	960	558	1049	601
Cmpd8vsUn	OxvsCmpd8	3171	413	1114	477	1167
Cmpd8vsUn	OxvsLor	3165	615	1041	557	952
Cmpd8_OxvsCmpd8	Lor_OxvsCmpd8	3174	1108	456	1257	353
Cmpd8_OxvsCmpd8	Lor_OxvsCmpd8_Ox	3178	823	722	975	658
Cmpd8_OxvsLor	Cmpd8_OxvsCmpd8	3185	936	545	991	713

Cmpd8_OxvsLor	Lor_OxvsCmpd8	3173	730	840	698	905
Cmpd8_OxvsLor	Lor_OxvsCmpd8_Ox	3183	551	999	535	1098
Cmpd8_OxvsLor	Lor_OxvsLor	3175	927	639	911	698
Cmpd8_OxvsOx	Cmpd8vsLor	3171	717	910	680	864
Cmpd8_OxvsOx	Cmpd8_OxvsCmpd8	3183	1012	469	1122	580
Cmpd8_OxvsOx	Cmpd8_OxvsLor	3190	961	688	910	631
Cmpd8_OxvsOx	Lor_OxvsCmpd8	3171	873	696	895	707
Cmpd8_OxvsOx	Lor_OxvsCmpd8_Ox	3182	716	833	757	876
Cmpd8_OxvsOx	Lor_OxvsLor	3168	824	743	850	751
Cmpd8_OxvsOx	Lor_OxvsOx	3176	1105	449	1156	466
Cmpd8_OxvsOx	OxvsLor	3185	415	1260	352	1158
Cmpd8_OxvsUn	Cmpd8vsLor	3174	610	1020	661	883
Cmpd8_OxvsUn	Cmpd8_OxvsCmpd8	3185	1096	383	1294	412
Cmpd8_OxvsUn	Cmpd8_OxvsLor	3192	971	678	1006	537
Cmpd8_OxvsUn	Cmpd8_OxvsOx	3190	1166	426	1256	342
Cmpd8_OxvsUn	Lor_OxvsCmpd8	3171	948	619	1059	545
Cmpd8_OxvsUn	Lor_OxvsCmpd8_Ox	3181	754	794	879	754
Cmpd8_OxvsUn	Lor_OxvsLor	3168	916	648	1031	573
Cmpd8_OxvsUn	Lor_OxvsOx	3168	1022	532	1145	469
Cmpd8_OxvsUn	Lor_OxvsUn	3178	1119	409	1284	366
Cmpd8_OxvsUn	OxvsCmpd8	3173	618	917	763	875
Cmpd8_OxvsUn	OxvsLor	3176	588	1076	608	904
LorvsUn	Cmpd8vsLor	3172	373	1249	409	1141
LorvsUn	Cmpd8vsUn	3175	1055	511	1163	446
LorvsUn	Cmpd8_OxvsCmpd8	3166	920	542	1119	585
LorvsUn	Cmpd8_OxvsLor	3179	570	1058	604	947
LorvsUn	Cmpd8_OxvsOx	3174	1027	549	1118	480
LorvsUn	Cmpd8_OxvsUn	3183	1107	380	1294	402
LorvsUn	Lor_OxvsCmpd8	3160	992	568	1092	508
LorvsUn	Lor_OxvsCmpd8_Ox	3163	930	615	1044	574
LorvsUn	Lor_OxvsLor	3167	807	751	903	706
LorvsUn	Lor_OxvsOx	3159	1095	453	1207	404
LorvsUn	Lor_OxvsUn	3172	1145	377	1297	353
LorvsUn	OxvsCmpd8	3164	605	926	736	897
LorvsUn	OxvsLor	3173	362	1293	367	1151
Lor_OxvsCmpd8	Lor_OxvsCmpd8_Ox	3175	1294	268	1326	287
Lor_OxvsLor	Cmpd8_OxvsCmpd8	3161	986	477	1123	575
Lor_OxvsLor	Lor_OxvsCmpd8	3166	1279	299	1291	297
Lor_OxvsLor	Lor_OxvsCmpd8_Ox	3170	1200	361	1232	377
Lor_OxvsOx	Cmpd8vsLor	3154	484	1135	473	1062
Lor_OxvsOx	Cmpd8_OxvsCmpd8	3161	984	481	1130	566
Lor_OxvsOx	Cmpd8_OxvsLor	3167	708	924	688	847
Lor_OxvsOx	Lor_OxvsCmpd8	3166	1240	337	1265	324
Lor_OxvsOx	Lor_OxvsCmpd8_Ox	3173	1177	384	1223	389
Lor_OxvsOx	Lor_OxvsLor	3164	1137	438	1162	427

Lor_OxvsOx	OxvsLor	3169	444	1227	395	1103
Lor_OxvsUn	Cmpd8vsLor	3160	473	1150	489	1048
Lor_OxvsUn	Cmpd8_OxvsCmpd8	3161	1029	430	1207	495
Lor_OxvsUn	Cmpd8_OxvsLor	3171	754	875	765	777
Lor_OxvsUn	Cmpd8_OxvsOx	3170	966	607	1035	562
Lor_OxvsUn	Lor_OxvsCmpd8	3168	1289	288	1342	249
Lor_OxvsUn	Lor_OxvsCmpd8_Ox	3170	1175	385	1245	365
Lor_OxvsUn	Lor_OxvsLor	3169	1201	374	1256	338
Lor_OxvsUn	Lor_OxvsOx	3169	1293	271	1359	246
Lor_OxvsUn	OxvsCmpd8	3159	757	772	865	765
Lor_OxvsUn	OxvsLor	3164	600	1059	581	924
OxvsCmpd8	Cmpd8vsLor	3170	652	984	660	874
OxvsCmpd8	Cmpd8_OxvsCmpd8	3174	859	602	1042	671
OxvsCmpd8	Cmpd8_OxvsLor	3173	805	830	807	731
OxvsCmpd8	Cmpd8_OxvsOx	3181	390	1182	451	1158
OxvsCmpd8	Lor_OxvsCmpd8	3169	945	614	1030	580
OxvsCmpd8	Lor_OxvsCmpd8_Ox	3166	862	682	951	671
OxvsCmpd8	Lor_OxvsLor	3157	914	646	984	613
OxvsCmpd8	Lor_OxvsOx	3168	608	938	685	937
OxvsCmpd8	OxvsLor	3175	1176	496	1132	371
OxvsLor	Cmpd8vsLor	3169	1148	472	1046	503
OxvsLor	Cmpd8_OxvsCmpd8	3166	673	795	713	985
OxvsLor	Cmpd8_OxvsLor	3179	1102	528	990	559
OxvsLor	Lor_OxvsCmpd8	3161	707	855	647	952
OxvsLor	Lor_OxvsCmpd8_Ox	3165	697	846	659	963
OxvsLor	Lor_OxvsLor	3167	882	676	842	767
OxvsUn	Cmpd8vsLor	3163	679	943	732	809
OxvsUn	Cmpd8vsUn	3171	865	698	990	618
OxvsUn	Cmpd8_OxvsCmpd8	3164	863	601	1072	628
OxvsUn	Cmpd8_OxvsLor	3174	866	765	912	631
OxvsUn	Cmpd8_OxvsOx	3183	597	975	703	908
OxvsUn	Cmpd8_OxvsUn	3180	938	550	1138	554
OxvsUn	LorvsUn	3174	863	637	1051	623
OxvsUn	Lor_OxvsCmpd8	3159	924	639	1033	563
OxvsUn	Lor_OxvsCmpd8_Ox	3164	838	707	964	655
OxvsUn	Lor_OxvsLor	3160	935	626	1046	553
OxvsUn	Lor_OxvsOx	3169	725	822	846	776
OxvsUn	Lor_OxvsUn	3172	971	551	1137	513
OxvsUn	OxvsCmpd8	3173	1050	496	1175	452
OxvsUn	OxvsLor	3178	1003	670	1004	501

Table 4 Dataset 1 information

X-axis	Y-axis	Tot. genes	Q1	Q2	Q3	Q4
CPT	DEL	2457	898	178	993	320
CPT	LZD	2457	873	144	1033	353
DEL	LZD	2457	817	194	1124	256
VAN	CPT	2457	855	378	626	548
VAN	DEL	2457	764	319	683	630
VAN	LZD	2457	745	280	727	657

Table 5 Dataset 3 information

X-axis	Y-axis	Tot. genes	Q1	Q2	Q3	Q4
Glu	Suc	1814	529	373	528	384
Glu	Xyl	1812	445	430	470	467
Lac	Glu	1814	374	539	389	512
Lac	Suc	1850	134	790	163	763
Lac	Xyl	1849	749	139	814	147
Suc	Xyl	1848	196	692	233	727

Table 6 Dataset 4 information

X-axis	Y-axis	Tot. genes	Q1	Q2	Q3	Q4
Ag_t03	AgXX_t03 3025	1031	443	1080	471	
Ag_t03	V2A_t03	3025	1082	464	1059	420
Ag_t90	AgXX_t90	3025	1028	334	1230	433
Ag_t90	V2A_t90	3025	1116	305	1259	345
V2A_t03	AgXX_t03	3025	1040	434	1045	506
V2A_t90	AgXX_t90	3025	1054	308	1296	367

5 Colors of the scorecards

Table 8 User-defined color scheme of the scorecards applied to each dataset

	ROIs of the Standard[1]					ROIs of the Full[2]		
Dataset	A	B	C	D	E	M	S	R
1	green	teal	blue	red	magenta	orange	gold	plum
2	goldenrod	limegreen	dodgerblue	sandybrown	crimson	darkcyan	indigo	orangered
3	tomato	darkorange	olivedrab	powderblue	hotpink	peru	khaki	lavender
4	indigo	darkorange	chocolate	lightcoral	forestgreen	mintcream	pink	bisque

Note: The color names are from Matplotlib library [1], and follow the CSS color table at https://matplotlib.org/stable/gallery/color/named_colors.html.

[1]Colors of the regions of interest inside each quadrant of the standard scorecard.

[2]Additional colors of the full scorecard for the inner regions of interest of each quadrant.

6 Scorecard software processing information

The scorecard could be a valuable method for visualizing relative fold-change comparisons: two treatments are compared to a control group by plotting their respective log fold-changes. The x-axis represents the log fold-change of treatment A versus the control, while the y-axis shows the log fold-change of treatment B versus the control. The axes of the scorecard depict the fold-change, which is the magnitude of changes between different conditions and is often computed considering the shrinkage estimate for dispersion that stabilizes the negative components [2], a measure that focuses on the size of the effect between conditions being evaluated together. It offers a complete set of analysis tools to assess the input data based on relative fold-change differences and statistics when multiple experimental conditions are examined.

The structure and nomenclature of the scorecard follow these characteristics:

- Axes Representation: Each axis of the Cartesian plane represents the fold-change in gene expression levels between two conditions: typically, "Treatment X" versus "Control" and "Treatment Y" versus "Control". By thresholding the bidimensional points, one could create a series of regions of interest to track the behavior of common genes.
- Quadrants: The axes divide the plot into four quadrants, each representing a different combination of upregulation and downregulation values across conditions:
  - Quadrant I (Q1): contains genes upregulated in both comparisons (i.e., upregulated in treatment X and treatment Y).
  - Quadrant II (Q2): includes genes upregulated in one comparison and downregulated in the other (i.e., upregulated in treatment Y and downregulated in treatment X).
  - Quadrant III (Q3): reports genes downregulated in both comparisons (i.e., genes downregulated in treatment X and treatment Y).
  - Quadrant IV (Q4): contains genes downregulated in one comparison and upregulated in the other (i.e., genes downregulated in treatment Y and upregulated in treatment X).

  Each quadrant is further subdivided into regions of interest based on user-defined thresholds (regions A, B, C, D, and E in the standard scorecard, adding M, R, and S zones for the full scorecard).
- Data Points: Each point on the plot represents a gene. The position of a point reflects the relative fold change in expression for that gene under each experimental condition. In addition, the scorecard only includes a textual description of the corresponding gene if the data point is statistically significant in both experiments.
- Color Coding: The colors of data points inside each region of interest can be user-defined. The same color scheme is applied to all additional graphs derived from the scorecard. Supplementary materials section **??** declares the colors employed during the analysis of each dataset.

This section contains a summary of the software library, which is useful as a reference for users interested in the scorecard workflow. Moreover, it helps understand the analyses presented throughout the manuscript. The scorecard functions included

in the library should automatically handle all computations, requiring the user to adjust only the parameters to obtain personalized outcomes.

Setting Parameters

The scorecard is imported, declaring the library in the preamble of the script:

```
import scorecard_functions
```

Afterward, one could create the parameter set for adjusting the scorecard features as follows:

```
param_dict=scorecard_functions.generate_parameters()
```

The variable `param_dict` is initialized with the default parameters, and users can modify them according to their preferences. For the parameter list and meaning, one could reference the GitHub repository or call the internal support of the scorecard library:

```
scorecard_functions.help()
```

Loading Data

Data can be loaded in CSV format containing precomputed fold-change and adjusted p-values for each gene through the function:

```
df=scorecard_functions.data_loading(param_dict)
```

The expression levels are stored in computer memory as Pandas DataFrame (`df` variable).

Creating ROIs and color Legend

It could be a good practice to create a legend of the color codes and the regions of interest for future reference by typing:

```
scorecard_functions.scorecard_legend(param_dict)
```

The legend is saved in the preselected folder as a separate image.

Scorecard calculations and Plots

Each quadrant of the scorecard is computed separately and saved as an image by calling the function:

```
scorecard_functions.scorecard(df,param_dict)
```

Given that each quadrant is saved as a single image, this trick ensures better visualization of the scatterplot in the case of packed entries.

Scorecard Assembly

By calling the code, the scorecard is assembled using the information of each quadrant:

```
scorecard_functions.reconstruct_scorecard(df,param_dict)
```

All quadrants are consolidated in the scorecard image. Three types of scorecards can be created by tuning the inclusion level.

Multiple Comparisons

A graph producing a general overview of all wet-lab experimental conditions might aid in the interpretation of results. A circular graph can be saved by calling the following function, which displays all compared experiments aligned along the radii.

The input is the name of the folder enclosing the subfolders that contain the different scorecards:
`scorecard_functions.multiple_view(main_folder)`
Additionally, all comparisons belonging to one quadrant of the scorecard could be saved as separate images.

Visualizations
Other functions applicable to the folder containing all the previously created scorecards produce supplementary graphs to investigate all aspects of the data extracted inside the regions of interest.
To build volcano plots and highlight genes or entries belonging to the regions of interest of the scorecard, one could type:
`scorecard_functions.make_volcano(main_folder)`
Single-entry analysis of the genes identified by the scorecard could involve the following line of code. The detected genes in each experimental condition will be reported as paired bar plots:
`scorecard_functions.multiple_bars(main_folder)`
Selected genes could be ranked by their absolute (unsigned) expression levels and plotted as a circular bar plot:
`scorecard_functions.ranking_bars(main_folder)`
To summarize and count the number of entries/genes identified by the scorecard, the following function can be used to write a textual report:
`scorecard_functions.count_frequencies(main_folder)`
Additionally, the number of entries/genes extracted by the scorecard can be visualized as a heatmap:
`scorecard_functions.Quadrants_heatmap(main_folder)`

Final Report
Detailed textual reports on the occurrence of common or rare (present in only one scorecard among all those created) genes could be saved with the subsequent line of code:
`scorecard_functions.common_entries(main_folder)`
A Cartesian graph displaying how common genes modify their expression level over time or experiments on the scorecard quadrants and areas of interest, could be created by the function:
`scorecard_functions.track_over_exper(main_folder)`
A CSV file or Excel table could be created by ranking all the genes identified by the scorecard, ordering them by the absolute difference of their expression:
`scorecard_functions.largest_diff(main_folder)`

All images built by the software library are at a resolution of 300 dpi, and could be considered "publication-ready". The scorecard software library has been released for the general public [3]: the analysis performed and the graphs shown in the present text could be reproduced by readers with minimum Python programming language experience. In the event that it is required, a four-way plot using a single fold-change

threshold can be created through the scorecard functions. However, producing a four-way plot limits the functionalities and outputs of the software, which is optimized to exploit the standard or full scorecard outcomes.

In summary, the Python library creates the following images:

- A legend of the scorecard to identify group naming conventions and the color applied to the regions of interest
- The full or standard scorecard:
  - A scorecard to detect extreme values only (e.g., right panel of Fig. **??**), in the text quoted as the "standard scorecard", or a scorecard including both extreme and mildly changing values, each channeled in a specific group (e.g., left panel of Fig. **??**), called the "full scorecard".
- Each quadrant of the scorecard is saved as an additional picture to allow zooming and inspecting the entries.
- An image of all comparisons between experimental conditions computed through the scorecard, in the form of a circular graph.
- Volcano plots of each experimental condition, with graphs arranged side-by-side.
- Expression level visualization as an annotated bar plot of all entries identified by the scorecard falling inside each quadrant. The bars follow the color scheme the user sets to quickly recap the regions of interest.
- Identified genes ranked by their absolute expression levels, arranged as a circular bar plot.
- A heatmap reporting the number of entries identified in each quadrant and region of interest (also saved separately as a CSV or Excel table).
- A bar plot of the genes that most frequently appeared in all the comparisons performed (in the background, the software automatically saves Excel or CSV files listing common and unique entries).
- Visualization of the recurring genes across experiments over the scorecard regions.

Complementary information is stored on the hard drive in the form of textual reports summarizing the results of the analyses carried out, CSV or Excel tables saved for later interpretation, and JSON archives, which make it convenient to import the scorecard results into other analysis pipelines.

7 A tutorial on a toy dataset

Inside the "examples" folder of the GitHub repository, a CSV (Comma-Separated Values) file simulating (log_2) fold change and p-values for ten hypothetical experimental conditions has been uploaded as a toy example to test scorecard library functionalities. By downloading the CSV file and cloning the scorecard repository locally, one can run the following code to obtain the full spectrum of images and reports built by the scorecard. The dataset was created artificially and is not constituted by computations on biological samples. Each column contains simulated fold changes and p-values representing ten bacteria cultured in the laboratory and exposed to a particular experimental condition (versus a control or baseline). The ten bacterial cultures could be

compared to each other. Gene IDs are alphanumeric codes built by randomly mixing numbers and letters. The attributes of each column could be interpreted as post-processed RNA-seq data from bacteria cultured to study the set of RNA transcripts produced by bacteria under theoretical treatment or laboratory conditions. The synthetic dataset presumed ten bacteria species to be included in this experimental data collection (Table 9):

Table 9 Fictitious bacteria present in the toy dataset.

Abbreviations	Bacteria
MRSA	Methicillin-Resistant Staphylococcus aureus
VRE	Vancomycin-Resistant Enterococcus
EHEC	Enterohemorrhagic Escherichia coli
VRSA	Vancomycin-Resistant Staphylococcus aureus
CRE	Carbapenem-Resistant Enterobacteriaceae
MDR_TB	Multidrug-Resistant Mycobacterium tuberculosis
XDR_TB	Extensively Drug-Resistant Mycobacterium tuberculosis
GAS	Group A Streptococcus
GBS	Group B Streptococcus
NTHi	Nontypeable Haemophilus influenzae

```
import scorecard_functions as sf

name_list=["MRSA","VRE","EHEC","VRSA",
    "CRE","MDR_TB","XDR_TB","GAS","GBS","NTHi"]
unq = sf.identified_comparisons(name_list)

sf.help()
# Current folder (replace with correct address)
mydir="D:/My_Folder/Example_Dataset"
param_dict=sf.generate_parameters()
print(param_dict) # standard parameters
# F.C. lower threshold
param_dict["th_fold_change"]=3
# Input directory where the toy dataset resides
param_dict["base_dir"]=mydir
# Toy dataset available on GitHub repository
param_dict["filename"]="ex_data.csv"
# Baseline condition name
param_dict["Control name"]="Ctrl"
# Column name of the gene symbols
param_dict["gene_name"]="ID"
param_dict["CSV delimiter"]=","
param_dict["Scorecard title"]="Toy Example"
# F.C. upper threshold (aka, multiplication factor of the lower)
param_dict["multiplication factor"]=1.5
# p-value for thresholding genes
param_dict["th_significance"]=0.01
```

```
# Prepare the full scorecard
param_dict["incl aver"]=True
# Output folder of the analysis
param_dict["save_dir"]=mydir
for ind, val in enumerate(unq):
    e_x,e_y=val[0],val[1]
    print("Num ",ind+1," of ",len(unq)," [",e_x," - ",e_y,"]")
    param_dict["FC cond x"]=e_x+"_FoldCh" # Scorecard x-axis
    param_dict["FC cond y"]=e_y+"_FoldCh" # Scorecard y-axis
    param_dict["padj cond x"]=e_x+"_AdjP"
    param_dict["padj cond y"]=e_y+"_AdjP"
    param_dict["Treatment1 name"]=e_x # Treatment 1
    param_dict["Treatment2 name"]=e_y # Treatment 2
    df=sf.data_loading(param_dict)
    sf.scorecard_legend(param_dict)
    # Intermediate data, individual quandrants
    sf.scorecard(df,param_dict)
# Actual scorecard creation
sf.reconstruct_scorecard(mydir,add_space=0.1)
# Radial plot of all exp. cond.
sf.multiple_view(mydir,fs_size=6,single_quadr=True)
sf.make_volcano(mydir) # Paired volcano plots
# F.C. bars
sf.multiple_bars(mydir, try_adj_test=False,
                 text_adj_x=0.0,text_adj_y=0.0)
# Ranking genes by their expr. levels
sf.ranking_bars(mydir)
# Occurrences counts
sf.count_frequencies(mydir)
# Common genes across experiments
sf.common_entries(mydir,do_excel=True,linewidth=0.2,fs_size=1.25)
# Report the number of instances inside each ROI
sf.Quadrants_heatmap(mydir)
# Identify genes across experiments
sf.track_over_exper(mydir,is_time=False)
# Rank genes from all experiments by magnitude
sf.largest_diff(mydir)
```

References

[1] Hunter, J.D.: Matplotlib: A 2d graphics environment. Computing in Science & Engineering **9**(3), 90–95 (2007) https://doi.org/10.1109/MCSE.2007.55

[2] Love, M.I., Huber, W., Anders, S.: Moderated estimation of fold change and dispersion for rna-seq data with deseq2. Genome Biology (2014) https://doi.org/10.1186/s13059-014-0550-8

[3] Nascimben, M.: A scorecard to compare wet-lab experimental conditions (2024). https://doi.org/10.5281/zenodo.13808354 . https://github.com/m89p067/

Scorecard
